# Phosphorylated FOXQ1, a novel substrate of JNK1, inhibits sorafenib-induced ferroptosis by activating ETHE1 in hepatocellular carcinoma

**DOI:** 10.1038/s41419-024-06789-1

**Published:** 2024-06-05

**Authors:** Yiwei Liu, Ke Shao, Wendong Yang, Qi Shen, Mengru Lu, Zhiying Shao, Sufang Chu, Yuming Wang, Xuehao Wang, Xiaofeng Chen, Jin Bai, Xiaofeng Wu

**Affiliations:** 1 Hepatobiliary Center, The First Affiliated Hospital of Nanjing Medical University; Key Laboratory of Liver Transplantation, Chinese Academy of Medical Sciences; NHC Key Laboratory of Living Donor Liver Transplantation (Nanjing Medical University), Nanjing, Jiangsu Province China; 2 Department of General Surgery, The People’s Hospital of Rugao, Affiliated Rugao Hospital of Nantong University, Nantong, Jiangsu Province China; 3 Cancer Institute, Xuzhou Medical University, Xuzhou, Jiangsu Province China; 4 Department of Oncology, The First Affiliated Hospital of Nanjing Medical University, Nanjing, Jiangsu Province China; 5 Center of Clinical Oncology, Affiliated Hospital of Xuzhou Medical University, Xuzhou, Jiangsu Province China

**Keywords:** Cancer therapeutic resistance, Cancer

## Abstract

Hepatocellular carcinoma (HCC) is a highly heterogeneous and malignant cancer with poor overall survival. The application of sorafenib is a major breakthrough in the treatment of HCC. In our study, FOXQ1 was significantly overexpressed in sorafenib-resistant HCC cells and suppressed sorafenib-induced ferroptosis. We found that phosphorylation of FOXQ1 at serine 248 is critical for the suppression of sorafenib-induced ferroptosis. Furthermore, as the upstream phosphorylation kinase of FOXQ1, JNK1, which is activated by sorafenib, can directly phosphorylate the serine 248 site of FOXQ1. Then, the phosphorylated FOXQ1 got a high affinity for the promoter of ETHE1 and activates its transcription. Further flow cytometry results showed that ETHE1 reduced intracellular lipid peroxidation and iron levels. Collectively, our study implicated the JNK1-FOXQ1-ETHE1 axis in HCC ferroptosis induced by sorafenib, providing mechanistic insight into sensitivity to sorafenib therapy of HCC.

## Introduction

Hepatocellular carcinoma (HCC) is one of the most common neoplasms and is the third leading cause of cancer-related death with an increasing incidence [1]. Due to the lack of early detection methods, most patients are already at an advanced stage at the time of initial diagnosis [2]. Sorafenib, a small-molecule multikinase inhibitor, is the first systemic medicine approved for HCC and is considered as a first-line treatment for advanced HCC. However, the curative effect of sorafenib remains limited and patients are prone to drug resistance. It is undeniable that tremendous progress has been made in HCC treatment over the past decades, but the prognosis of HCC patients is still poor [3]. Therefore, it is necessary to find reliable biomarkers to predict the treatment effect of HCC and develop new treatment strategies.

Ferroptosis is a newly identified form of iron-dependent regulated cell death (RCD) characterized by the accumulation of lipid reactive oxygen species (ROS) [4, 5]. To date, ferroptosis has been shown to be effective in killing various cancer cells, including HCC cells. A large number of studies have reported that sorafenib can induce ferroptosis by inhibiting system Xc-[6]. Notably, sorafenib-induced ferroptosis is an effective mechanism for inducing cell death independent of its kinase inhibitory function in HCC, and plays an integral anticancer role in the treatment of HCC [7]. However, the effect of sorafenib in clinical use is unsatisfactory. With sorafenib treatment, the median survival time of HCC patients can only be prolonged 3-5 months. Therefore, how to improve the sensitivity of HCC to sorafenib and improve the effectiveness of anticancer treatment is still an urgent unmet need for patients with advanced HCC.

Forkhead Box Q1 (FOXQ1) is a newly identified member of the forkhead (FOX) transcription factor family. It functions as a transcription factor with a conserved FOX DNA-binding domain and has been shown to play an important role in EMT, invasion and metastasis in many cancers, including breast cancer, non-small cell lung cancer, bladder cancer, colorectal cancer and HCC [8–12]. Recent studies have shown that FOXQ1 is closely related to the treatment resistance of various tumors. It promotes the stemness and radioresistance of colorectal cancer cells by activating SIRT1 expression and enhancing β-catenin expression [13]. Upregulation of FOXQ1 by circular RNA CRIM1 promotes metastasis and docetaxel chemoresistance in nasopharyngeal carcinoma [14]. Through FOXQ1/Twist1/PDGFR axis, FOXQ1 can also promote the stemness and chemoresistance of mammary epithelial cells [15]. Although an oncogenic role of FOXQ1 in HCC has been reported in several studies [12, 16, 17], the role of FOXQ1 in the resistance of HCC to treatment is unknown, especially how it is related to HCC cell ferroptosis.

Here, we conducted a novel mechanistic study and elucidated how FOXQ1 was involved in sorafenib resistance. We observed increased expression of FOXQ1 in sorafenib-resistant cells, and high expression of FOXQ1 led to a poor prognosis for sorafenib-treated patients. Mechanistically, phosphorylation of FOXQ1 by JNK1 transcriptionally activates the expression of ETHE1, thereby protecting HCC cells from sorafenib-induced ferroptosis.

Our study demonstrates that targeting ferroptosis may be a promising new strategy to increase sensitivity to sorafenib therapy in HCC and that FOXQ1 may be a potential therapeutic target.

## Materials and methods

### Cell culture and transfection

All cell lines were purchased from the American Type Culture Collection (ATCC) and maintained at 37 °C with 5% CO_2_. PLC/PRF/5、SK-Hep1 and HEK293T cells were cultured in Dulbecco’s modified Eagle’s medium supplemented (KeyGEN bio, KGL1206-500) with 10% fetal bovine serum (Invitrogen). The concentration of Sorafenib is 10 μM. Transfections were performed according to the manufacturer’s instructions with lipofectamine 2000 (Invitrogen) or silentfect (Bio-rad).

### Plasmids and siRNAs

pcDNA3.1-FOXQ1, pGEX-6p1-FOXQ1, PGL3-FOXQ1 promoter, and pLKO.1-shETHE1 were synthesized and sequenced by Genewiz company. For pLenti-CMV-GFP-FOXQ1 and pcDNA3.1-JNKs, We clone FOXQ1 into pLenti-CMV-GFP with BamH I and EcoR I as restriction sites and clone JNKs into pcDNA3.1 with BamH I and EcoR I as restriction sites respectively. All the above plasmids have been verified by sequencing. All siRNAs were synthesized by Genepharma company.

### Lentiviral particle production and infection

Lentiviral vectors (10 μg) were transfected with psPAX2 (7.5 μg) and pMD2G (2.5 μg) into HEK293T cells with Lipofectamine. Supernatants were collected at 48 and 72 h after transfection and filtered through 0.45 μm pore size filtration. For transduction, 3 × 10^5^ cells per well were seeded in six-well plates and infected with 1 mL per well of medium containing lentivirus. The medium was removed 24 h later and replaced with fresh medium containing 2 μg/mL puromycin. Cells were selected for about 2 weeks to generate stable cell lines.

### Antibodies and chemicals

The following antibodies were used in this study at the indicated dilution for western blot (WB) analysis, immunoprecipitation, and Immunohistochemistry (IHC): Flag (66008-4-Ig Proteintech 1:10,000 for WB), ACSL4 (22401-1-AP Proteintech 1:2000 for WB, 1:100 for IHC), FOXQ1 (23718-1-AP Proteintech 1:1000 for WB), P-FOXQ1 (S248) (Huabio 1:500 for WB), ETHE1 (27786-1-AP Proteintech 1:1000 for WB, 1:200 for IHC), Tubulin (66031-1-Ig Proteintech 1:50,000 for WB), GAPDH (60004-1-Ig Proteintech 1:100,000 for WB), Phospho-SAPK/JNK (Thr183/Tyr185) (#4668 S Cell Signaling Technology 1:1000 for WB), PARP1 (ab191217 Abcam 1:1000 for WB), JNK1 (ab199380 Abcam 1:2000 for WB), 4-HNE (ab48506 Abcam 1:25 for IHC), FOXQ1 (ab51340 Abcam 1:200 for IHC), Anti-Phosphoserine (ab9332 Abcam 1:1000 for WB), Anti-Phosphothreonine (ab9337 Abcam 1:1000 for WB), Anti-Phosphotyrosine (ab10321 Abcam 1:1000 for WB). Sorafenib (HY-10201) were obtained from MCE.

### Cell viability assay

Cell viability was evaluated by the cell counting kit-8 (CCK-8). Cells were seeded in 96-well plates (10,000 cells per well) and treated with DMSO, Sorafenib. The culture medium was replaced with 100 μL fresh medium with 10 μL of the CCK-8 solution for each well. And the plate was returned to the cell culture incubator for 4 h. Measure the absorbance at 450 nm using a microplate reader.

### Colony formation assay

For the colony formation assay, cells were digested, counted and diluted. Then 2000 cells were seeded onto each well of a six-well plate. After the cells adhered to the plate, sorafenib treatment was carried out. After 7–14 days, cells were fixed and stained with glutaraldehyde and crystal violet. After removing the crystal violet, wash the six-well plate with deionized water. At room temperature, the six-well plate with colonies was dried. Finally, the number of clones is counted. Each experiment was repeated three times.

### Co- immunoprecipitation (Co-IP) and western blotting

Cells were lysed by the buffer (50 mM Tris-HCl, pH 7.5, 0.5% NP-40, 150 mM NaCl, 2 mM EDTA) with protease inhibitor cocktail (Beyotime) for 30 min at 4 °C. Then the cellular lysates were centrifuged at 12,000 rpm for 10 min at 4 °C. 5% cellular extracts were used for input. For immunoprecipitation, 45 μl of 50% protein A or G agarose beads was added and the incubated with 4 μg antibodies for 7 h at 4 °C with constant rotation. 1 mg of protein was was then added and the incubation was continued for an additional 12 h; Beads were then washed four times with PBS. Between washes, the beads were centrifuged at 2000 rpm for 1 min at 4 °C for collection. The proteins were eluted from the beads by 1 × SDS-PAGE loading buffer and boiling for 10 min at 99 °C. The protein samples were resolved using SDS-PAGE gels and transferred onto nitrocellulose membranes. The phosphorylated band was blocked with 5% BSA (Beyotime, ST025), and the non-phosphorylated band was blocked with 5% skim milk (Beyotime, P0216) for 2 h at room temperature. Then, membranes were incubated with antibodies overnight at 4 °C followed by incubation with a secondary antibody for 2 h at room temperature. Western blotting Luminol reagent (Beyotime) and Biorad ECL machine were used for immunodetection and detection.

### Liquid chromatography-tandem mass spectrometry (LC-MS/MS)

Flag-FOXQ1 was transfected into PLC/PRF/5 cells, then purified and resolved by SDS-PAGE and the targeting bands were excised and digested with trypsin (Sigma Aldrich). The FOXQ1 peptide mixture was analyzed by HPLC-MS/MS (Q Exactive (Thermo Fisher), Easy-nLC 1000 (Thermo Fisher)). The mass-to-charge ratios of polypeptides and polypeptide fragments were collected according to the following method: 20 fragment spectra (MS2 scan) were collected after each full scan. Data was analyzed using Mascot2.2 software (Matrix Science, Boston, MA, USA).

### In vitro kinase assay

PLC/PRF/5 cells were transfected with FLAG-JNK1 plasmids. FLAG-JNK1 fusion proteins were then immuno-purified with anti-FLAG M2 attached with agarose and eluted by competitive elution using 3× Flag peptide. GST-FOXQ1 constructs were expressed in BL21 E. coli bacteria, and crude bacterial lysates were prepared by sonication in PBS in the presence of the protease inhibitor mixture. The lysates were then added to 50 μl of glutathione-Sepharose beads and incubated for 4 h at room temperature with constant rotation. The beads were washed three times with PBS. The FLAG-JNK1 and GST-FOXQ1 bond with beads were incubated with 1 mM ATP for 30 min at 30 °C in reaction buffer, followed by resuspended in 80 μL of 1 × SDS-PAGE loading buffer, and detected by western blot.

### Chromatin immunoprecipitation (ChIP)

ChIP was performed in PLC/PRF/5 cells as chip kit introduction (Beyotime, P2078). Briefly, 10^7^ cells were cross-linked with 1% formaldehyde for 10 min at 37 °C and quenched with 0.125 M glycine for 5 min at room temperature. Then the cells were collected and sonicated, pre-cleared. The lysates were incubated with 5 μg of antibody for 12 h at 4 °C. Protein A/G beads were added for additional 4 h at 4 °C. Complexes were washed with low salt, high salt and TE buffers, and the DNA was extracted and analyzed by real-time PCR or ChIP-seq according to the manufacturer’s instructions at the HUADA company. The primer sequences used for PCR were: qChIP-ETHE1 forward, 5′- CCTCCACCTCCCGAGT -3′ and qChIP-ETHE1 reverse, 5′- GTACGGACATTAGGGTCG-3′.

### Reporter assay

PLC/PRF/5 cells in 24-well plates were transfected with luciferase reporter, renilla, and indicated expression constructs. The amount of DNA in each transfection was kept constant by addition of empty vector. 36 h after transfection, the firefly and renilla luciferase were assayed according to the manufacturer’s protocol (Promega), and the firefly luciferase activity was normalized to that of renilla luciferase.

### RNA extraction, cDNA synthesis, and qPCR analysis

Total RNA was isolated with Trizol (Vazyme, R401-01) and used for cDNA synthesis by HiScript II 1st Strand cDNA Synthesis Kit (Vazyme, R212-01). Quantitative PCR was determined using LightCycler96 (Applied Biosystems) and calculated by means of the comparative Ct method (2^−ΔΔCt^) with GAPDH as an internal control.

### Lentiviral production and infection

pLenti-CMV-GFP-FOXQ1 was generated by subcloning the FOXQ1 fragment into the pLenti-CMV-GFP vector. pLKO.1-shETHE1 was generated by subcloning shRNA into the pLKO.1 vector. pLenti-CMV-GFP, pLenti-CMV-GFP-FOXQ1, pLKO.1, pLKO.1-shETHE1, together with psPAX2 and pMD2.G were co-transfected into the packaging cell line HEK293T. Viral supernatants were collected 72 h later, clarified by filtration, and concentrated by ultracentrifugation. The concentrated virus was used to infect SK-Hep1 cells (50% confluent) in a 60 mm dish with 5 μg/ml polybrene. Infected cells were selected by 2 μg/ml puromycin (Merck).

### Xenograph mouse model

All animal studies were approved by the Institutional Animal Care and Use Committee of Xuzhou Medical University. SK-Hep1 cells were infected with lentiviruses carrying vector + shCtrl (pLenti-CMV-GFP+pLKO.1), FOXQ1+shCtrl (pLenti-CMV-GFP-FOXQ1+pLKO.1), FOXQ1+shETHE1 (pLenti-CMV-GFP-FOXQ1+pLKO.1-shETHE1). To construct a subcutaneous tumor model, HCC cells were injected into nude mice via the left posterior flanks of 7-week-old immunodeficient female nude mice (*n* = 5 for each group). To monitor tumor development, tumor length and width were measured (tumor volume = 1/2(length × width^2^)). When the mouse volume reached 50mm^3^, the mice were divided into groups and injected intraperitoneally with sorafenib (30 mg/kg/d) for 2 weeks as report [18]. Mice were then sacrificed, and tumors were weighed, formalin-fixed and paraffin-embedded for Immunohistochemistry.

### Immunohistochemistry and Immunohistochemical score

Paraffin-embedded HCC samples were collected from 27 patients receiving sorafenib at the Hepatobiliary Center of the First Affiliated Hospital of Nanjing Medical University. This study was approved by the Ethics Committee of the First Affiliated Hospital of Nanjing Medical University, and all patients signed informed consent. It also complies with the provisions of the Declaration of Helsinki. For IHC staining, tissue slides were deparaffinized and rehydrated, followed by treated with 3% hydrogen peroxide. Microwave and 0.1 M citric sodium buffer (pH 6.0) were used for Antigen retrieval. Sections were then incubated with the primary antibody overnight at 4 °C. Antibody binding was detected with HRP-DAB kit (ZSGB-BIO, PV-9001/9002) and counterstained with haematoxylin. Images were acquired using a Olympus microscope and Olympus image software. Two pathologists were blinded to score the stained tissue sections separately using immunoreactive score of Remmele and Stegner systems (IRS).

Tissue microarrays were evaluated separately by two pathologists under blinded experimental conditions, and all resulting differences were resolved by discussion. The staining score was assessed by combining the percentage of cells and staining intensity according to the immunohistochemical score (IRS). Immunostaining intensity was scored as 0–3 (0, negative; 1, weak; 2, mild; 3, strong); the percentage of immunoreactive cells was scored as 1 (0–25%), 2 (26–50%), 3 (51–75%), and 4 (76–100%). The score of FOXQ1 were expressed as 1 (IRS: 0–4), 2 (IRS: 5-8), 3 (IRS: 9–12).

### Lipid peroxidation assay

Lipid peroxidation level was analyzed by flow cytometry using BODIPY-C11 dye. Cells were seeded in six-well plates at a density of 2.5 × 10^5^ per well and grown overnight. Cells were exposed to DMSO or Sorafenib for 24 h, and then incubated in 2 mL medium with 5 µM of BODIPY-C11 (Thermo Fisher, Cat# D3861) for 20 min in a cell culture incubator. After washing with PBS to remove excess dye, the cells were suspended in 500 µl of PBS and filtered through a 0.4 µm nylon mesh cell strainer before being analyzed using flow cytometry. At least 10,000 cells were examined per condition.

### Iron assay

Intracellular ferrous iron (Fe^2+^) was analyzed by flow cytometry using FerroOrange. Cells were seeded in six-well plates at a density of 2.5 × 10^5^ per well and grown overnight. Cells were treated with DMSO or sorafenib for 24 h. Trypsinize cells and collect in 1.5 mL tubes. Resuspend the cells in 1 mL HBSS containing 1 µM FerroOrange (Dojindo, Cat# F374) and return to the cell culture incubator for 30 min. The cellular fluorescence intensity of each sample was immediately determined by flow cytometry. At least 10,000 cells were analyzed for each condition.

### Kaplan-Meier analysis

In the Kaplan-Meier Plotter database (http://kmplot.com/analysis/), 30 liver cancer patients treated with sorafenib were divided into high expression group and low expression group according to the median expression of FOXQ1, and the prognosis of these 30 patients was analyzed by Kaplan-Meier survival analysis. The red line represents the survival curve of patients with high FOXQ1 expression, and the black line represents the survival curve of patients with low FOXQ1 expression.

### Nucleocytoplasmic separation experiments

The nuclear and cytoplasmic fractions were isolated using the Nuclear and Cytoplasmic Protein Extraction Kit (Beyotime, China). Cells were washed with PBS and scraped off with A cell scraper. For every 20 μL of cell precipitate, 200 μL of cytosolic protein extraction reagent A supplemented with Phenylmethanesulfonyl fluoride (PMSF) were added and then vortexed for 5 s followed by an ice bath for 10–15 min. 10 μL of cytosolic protein extraction reagent B was added and vortexed for 5 s followed by an ice bath for 1 min. After centrifugation, the supernatant was immediately aspirated into a precooled plastic tube, which was the extracted cytosolic protein. For precipitation, the remaining supernatant was completely aspirated, 50 μL of nuclear protein extraction reagent supplemented with PMSF were added, vortexed for 15 to 30 s and centrifuged for 10 min. The supernatant was immediately aspirated into a precooled plastic tube, which was the extracted nuclear protein.

### Statistical analysis

All data are presented as the mean ± SD in at least three biological experiments. Statistical analyses were conducted using GraphPad Prism 8 software, the comparison between the two groups of data was concluded by two-tailed Student’s *t* test. *p* < 0.05 considered the difference to be statistically significant.

## Results

### FOXQ1 is upregulated in sorafenib-resistant strains and suppresses sorafenib-induced ferroptosis in HCC

**Fig. 1:**
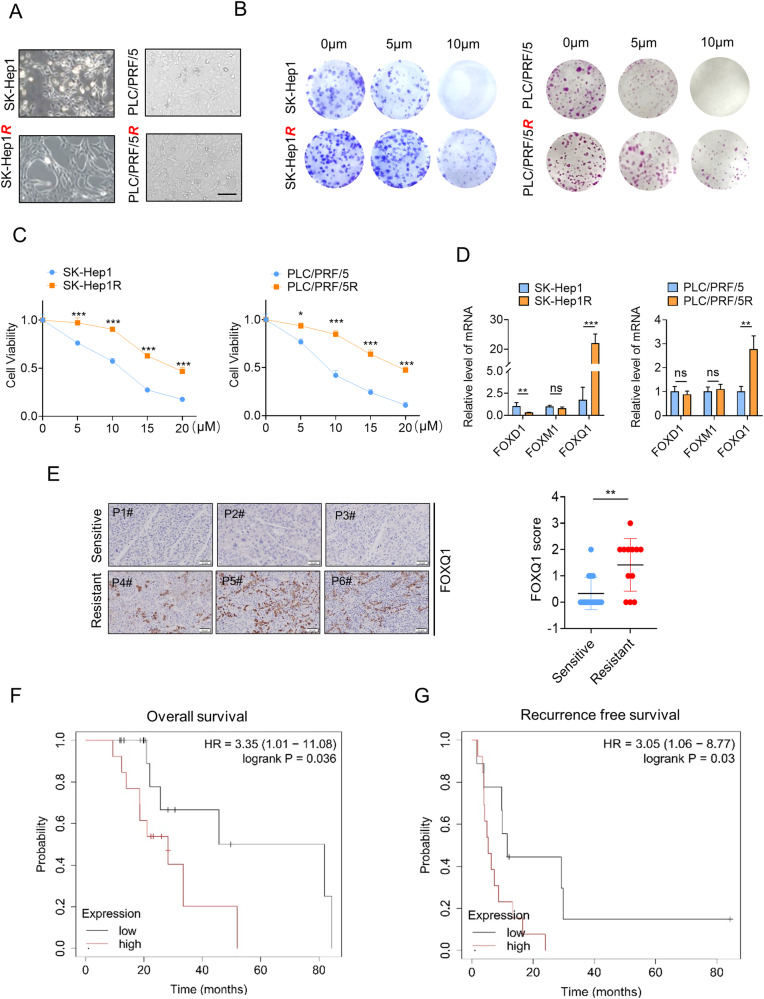
FOXQ1 is upregulated in sorafenib-resistant strains. **A** Images of naive SK-Hep1 and sorafenib-resistant SK-Hep1-R cells. **B** Evaluation of sorafenib treatment sensitivity of sorafenib-resistant cells and parental cells by clone formation assay. **C** The survival rate of sorafenib-resistant cells was evaluated by CCK-8. **D** The mRNA expression of FOXD1, FOXM1 and FOXQ1 in sorafenib-resistant cells and parental cells. **E** Representative IHC images of FOXQ1 expression are shown in sorafenib-sensitive and sorafenib-resistant clinical HCC samples (*t*-test) (*n* = 27), Scale bar= 20 μm. The relationship between FOXQ1 expression and prognosis in 30 sorafenib users was analyzed by the Kaplan-Meier Plotter database (http://kmplot.com/analysis/). (**F**: Overall survival, **G**: Recurrence free survival, The red line represents the survival curve of patients with high FOXQ1 expression, and the black line represents the survival curve of patients with low FOXQ1 expression. HR: Hazard ratio). **, *P* < 0.01; ***, *P* < 0.001.

We constructed sorafenib-resistant HCC cell lines, SK-Hep1R and PLC/PRF/5 R, and verified its sorafenib resistance through colony formation and CCK8 assays (Fig. 1A–C). It has been reported that 17 genes play a key role in digestive system tumors, of which three are in the FOX family (FOXD1, FOXM1, and FOXQ1) [19]. We explored the expression of these three FOX family members in the sorafenib-resistant cells. Compared to the parent SK-Hep1 and PLC/PRF/5 lines, only the expression of FOXQ1 was significantly increased (Fig. 1D). The postoperative pathological tissues of 27 HCC patients treated with sorafenib were collected, then the relationship between FOXQ1 immunohistochemical staining and the objective response rate of sorafenib treatment was analyzed, the results showed the expression of FOXQ1 in patients with sorafenib resistance was higher (Fig. 1E). In the Kaplan-Meier Plotter database (http://kmplot.com/analysis/), 30 liver cancer patients treated with sorafenib were divided into high expression group and low expression group according to the median expression of FOXQ1, and the prognosis of these 30 patients was analyzed by Kaplan-Meier survival analysis. The FOXQ1 high expression group had worse overall survival (OS), recurrence-free survival (RFS) (Fig. 1F, G) and disease-free survival (DFS) and Progression-free survival (PFS) (Fig. S1 A, B).

**Fig. 2:**
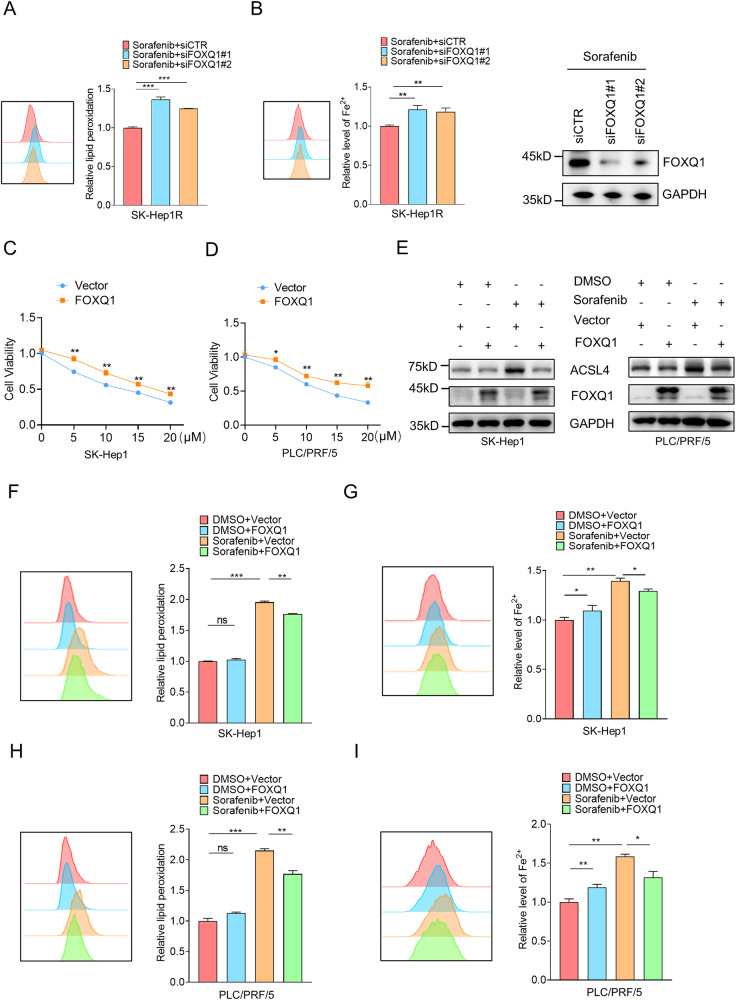
FOXQ1 suppresses sorafenib-induced ferroptosis in HCC. **A**, **B** Effect of FOXQ1 expression on iron content and lipid peroxidation in SK-Hep1-R cells were analyzed by flow analysis. **C**, **D** SK-Hep1 and PLC/PRF/5 cells were transiently transfected with Vector and FOXQ1, then CCK-8 was used to verify the effect of FOXQ1 expression on cell survival after sorafenib treatment. **E** The effect of FOXQ1 on the expression of ACSL4 in DMSO treatment group and sorafenib (10 μM) treatment group. **F–I** SK-Hep1 and PLC/PRF/5 cells were transiently transfected with Vector and FOXQ1, then the iron content and lipid peroxidation were assessed by flow cytometry after DMSO and sorafenib (10 μM). *P*-values were determined by Student’s *t* test. *, *P* < 0.05; **, *P* < 0.01; ***, *P* < 0.001.

Since sorafenib-induced ferroptosis plays an important role in the treatment of hepatocellular carcinoma [20], we explored whether FOXQ1 affects the therapeutic sensitivity of sorafenib by regulating ferroptosis. Knockdown of FOXQ1 in SK-Hep1R cells significantly increased lipid peroxidation levels and iron content (Fig. 2A, B), suggesting that FOXQ1 may affect sorafenib sensitivity by regulating sorafenib-induced ferroptosis. We then confirmed the relationship between FOXQ1 and sorafenib-induced ferroptosis. Overexpression of FOXQ1 attenuated the antitumor effect of sorafenib on hepatocellular carcinoma (Fig. 2C, D). After sorafenib treatment, overexpression of FOXQ1 reduced the expression of ACSL4, as well as the ferroptosis-related features such as intracellular lipid peroxidation level and iron content, while in the DMSO group, FOXQ1 had no obvious regulation on the above-mentioned ferroptosis features (Fig. 2E–I). These results suggest that FOXQ1 regulates the sensitivity of HCC cells to sorafenib treatment by inhibiting sorafenib-induced ferroptosis.

### Phosphorylation of serine 248 affects the regulation of ferroptosis by FOXQ1

**Fig. 3:**
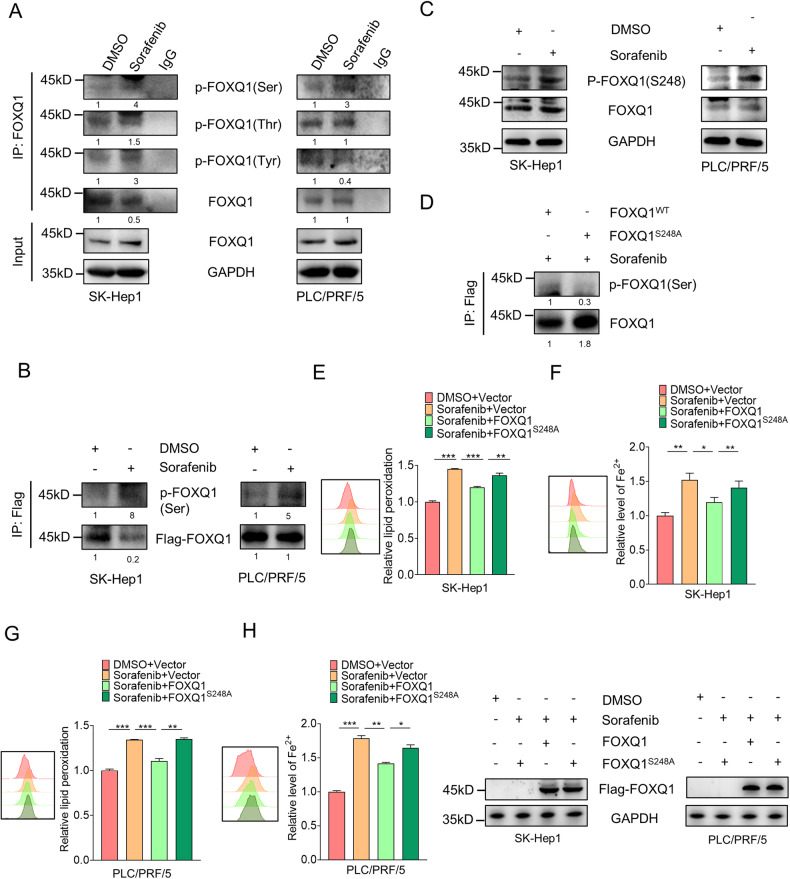
Phosphorylation of Serine 248 affects the regulation of ferroptosis by FOXQ1. **A** Effect of sorafenib (10 μM) on phosphorylation of FOXQ1 at serine, tyrosine and threonine sites. The numbers between the strips are grayscale values calculated by imageJ. **B** Effect of sorafenib (10 μM) treatment on the phosphorylation of Flag-FOXQ1 serine sites (Flag-FOXQ1 was transfected into cells instantaneously). **C** Effect of sorafenib (10 μM) on phosphorylation of FOXQ1 at serine 248 (S248). **D** The effect of the mutation at Serine 248 on the phosphorylation of FOXQ1. SK-Hep1 and PLC/PRF/5 cells were transiently transfected with Vector, FOXQ1 and FOXQ1S^248A^, then the iron content (**E**, **F**) and lipid peroxidation (**G**, **H**) were assessed by flow cytometry after DMSO and sorafenib (10 μM) treatment. *P* values were determined by Student’s *t*-test. *, *P* < 0.05; **, *P* < 0.01; ***, *P* < 0.001.

In previous experiments, we observed that FOXQ1 could inhibit ferroptosis induced by sorafenib. Sorafenib has been reported to affect protein phosphorylation [21], and the phosphorylation modification of transcription factors may change their transcriptional activity. Therefore, we speculate whether sorafenib regulates the transcriptional activity of FOXQ1 by affecting the phosphorylation of FOXQ1, and then targets ferroptosis-related genes. In order to verify the above conjecture, the phosphorylation of FOXQ1 at serine, threonine, and tyrosine sites were verified and only serine sites were detected to be triggered by sorafenib both in SK-Hep1 and PLC/PRF/5 (Fig. 3A). The protein modification mass spectrometry analysis showed that FOXQ1 could be phosphorylated at serine 248. Compared with the DMSO group, the phosphorylation of FOXQ1 serine 248 was significantly enhanced after sorafenib treatment (Fig. 3B, C), and mutation of serine 248 caused the decrease of FOXQ1 phosphorylation (Fig. 3D). Importantly, flow cytometry results showed that mutations at serine 248 reduced the inhibitory effect of FOXQ1 on intracellular lipid peroxidation and iron levels (Fig. 3E–H). The above results indicate that serine 248-phosphorylated FOXQ1 can inhibit the ferroptosis induced by sorafenib.

### JNK1 is an upstream phosphokinase involved in FOXQ1 phosphorylation

**Fig. 4:**
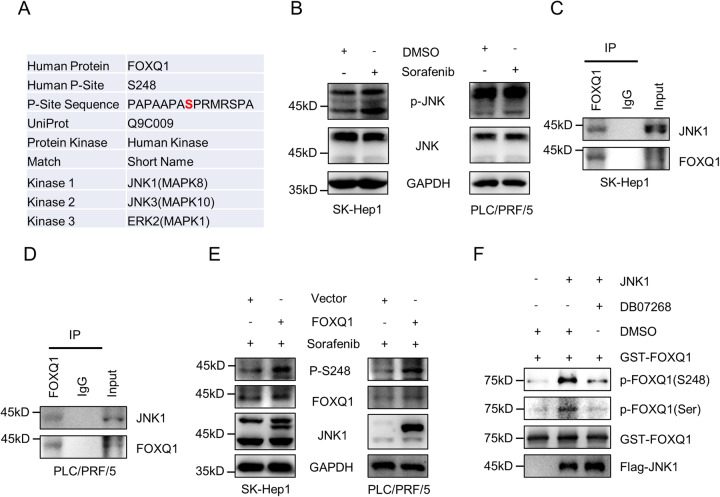
JNK1 is an upstream phosphokinase of FOXQ1 phosphorylation. **A** Possible upstream phosphorylated kinases of FOXQ1 predicted by PhosphoNET database (http://www.phosphonet.ca/). **B** Effect of sorafenib (10 μM) on JNK phosphorylation. **C, D** Detection of JNK1 binding to FOXQ1 by immunoprecipitation. **E** The effect of JNK1 expression on the phosphorylation of FOXQ1 at serine 248 (S248). **F** In vitro kinase assays verify the changes in phosphorylation of FOXQ1 when FOXQ1 was incubated with JNK1 or DB07258.

Since phosphorylation plays a key role in the regulation of ferroptosis by FOXQ1, we explored the upstream kinases of its phosphorylation. The PhosphoNET Database (http://www.phosphonet.ca/) predictions show that JNK1, JNK3 and ERK2 may be the potential upstream kinases of FOXQ1 serine 248 (Fig. 4A). Since we have proved that sorafenib induces the phosphorylation of FOXQ1, it should also positively regulate the upstream kinases of FOXQ1. Sorafenib has been reported to inhibit most kinases, including ERK [22]. We found that sorafenib can activate the phosphorylation of JNK. Interestingly, sorafenib mainly activates the phosphorylation of a subtype of JNK at 45 kDa. Since JNK3 is mainly located at 53 kDa and JNK1 is mainly located at 45 kDa (Fig. 4B), we speculate that JNK1 is the most likely upstream kinase that phosphorylates FOXQ1. Since a kinase needs to bind to its substrate to play a catalytic role, we investigated the interaction between FOXQ1 and JNK1 by Co-IP and found that JNK1 is able to bind to FOXQ1 (Fig. 4C, D). The overexpression of JNK1 significantly enhanced the phosphorylation of FOXQ1 at serine 248 (Fig. 4E). To confirm that JNK1 is the upstream kinase of FOXQ1, we conducted an in vitro kinase assay. The results showed that JNK1 can enhance the phosphorylation of FOXQ1 serine 248 in vitro and that the JNK1 inhibitor DB07268 can inhibit that (Fig. 4F). These results demonstrated that JNK1 is the upstream kinase that phosphorylates serine 248 of FOXQ1.

### JNK1 translocates into the nucleus after treatment with sorafenib

**Fig. 5:**
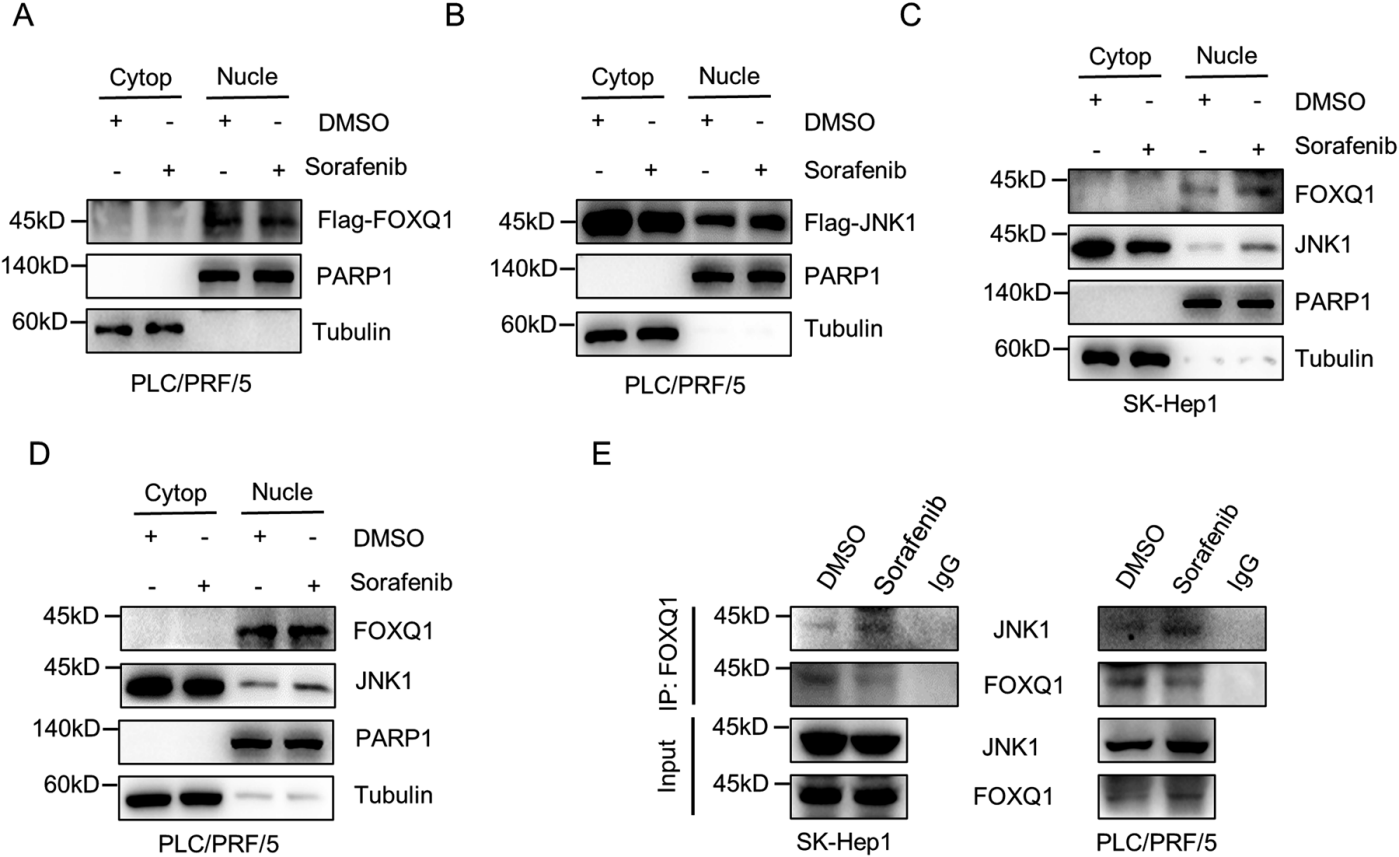
JNK1 enters the nucleus after treatment of sorafenib. Flag-FOXQ1 or Flag-JNK1 was overexpressed in PLC/PRF/5, and the expression of Flag-FOXQ1 (**A**) or Flag-JNK1 (**B**) in the nucleus and cytoplasm was verified after DMSO or sorafenib (10 μM) treatment for 24 h. **C**, **D** After DMSO or sorafenib (10 μM) treatment for 24 h, the expression of FOXQ1 and JNK1 in the nucleus and cytoplasm was verified. **E** After PLC/PRF/5 and SK-Hep1 were treated with DMSO or sorafenib (10 μM) for 24 h, the changes in the binding amount of JNK1 and FOXQ1 were detected by immunoprecipitation.

Translocation into the nucleus after phosphorylation is the key step for many proteins to exert their corresponding functions [23–25]. Therefore, we speculate that FOXQ1 also enters the nucleus after phosphorylation to affect transcriptional activity. Nucleocytoplasmic separation experiments were used to verify our conjecture, and the results showed that FOXQ1 was mainly localized in the nucleus, and no obvious nuclear translocation occurred after sorafenib treatment (Fig. 5A, C, D). Thus, we then speculated that it is the upstream kinase JNK1 rather than FOXQ1 that enter the nucleus. The results showed that after sorafenib treatment, JNK1 expression in the nucleus increased, while the part in the cytoplasm decreased, which indicated that JNK1 translocate from the cytoplasm into the nucleus after sorafenib treatment (Fig. 5B–D). More importantly, FOXQ1 bound more JNK1 in the sorafenib-treated group compared with the DMSO group (Fig. 5E). These results indicate that in response to sorafenib, JNK1 translocates into the nucleus to bind and phosphorylate FOXQ1.

### FOXQ1 activates ETHE1 transcription in HCC cells

**Fig. 6:**
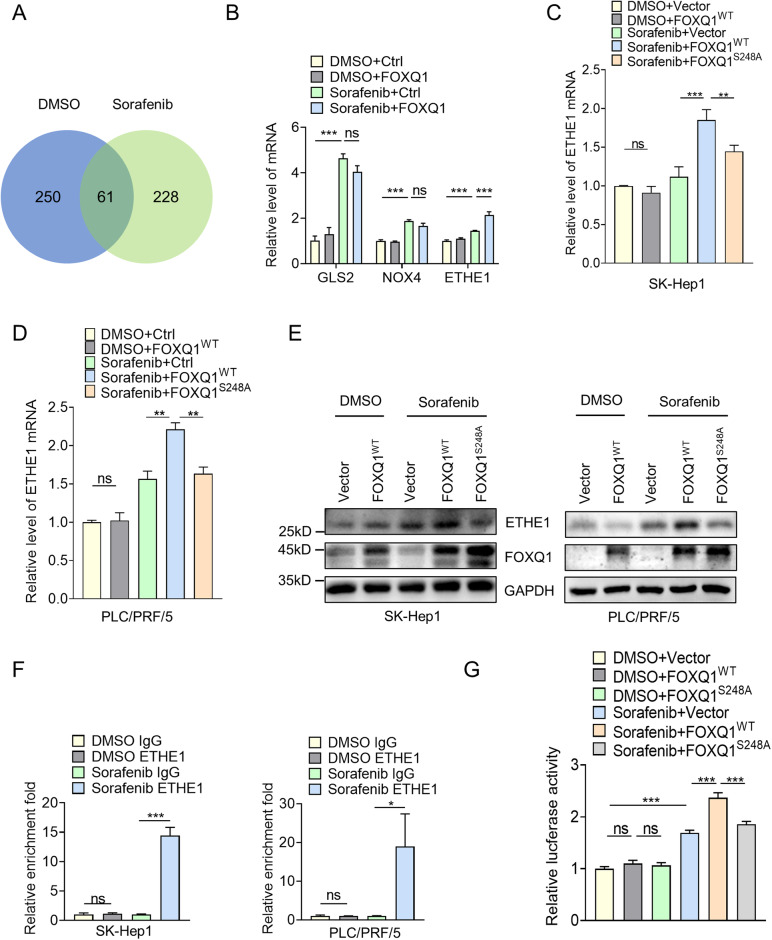
FOXQ1 activates the transcription of ETHE1 in HCC cells. **A** FOXQ1 ChIP-Seq was performed on PLC/PRF/5 cells treated with DMSO or sorafenib (10 μM) for 24 h, and the Venn diagram showed the ChIP-seq results of FOXQ1 in each group. **B** PLC/PRF/5 cells were transiently transfected with Vector and FOXQ1. Real-time quantitative polymerase chain reaction(qRT-PCR) to verify the effect of FOXQ1 expression on the expression of GLS2, NOX4 and ETHE1 after treatment with DMSO and sorafenib (10 μM). **C**–**E** SK-Hep1 and PLC/PRF/5 cells were transiently transfected with Vector, FOXQ1 and FOXQ1^S248A^, then the cells were treated with DMSO or sorafenib (10 μM) for 24 h, the effects of FOXQ1^WT^ and FOXQ1^S248A^ on the expression of ETHE1 were detected by real-time quantitative polymerase chain reaction (qRT-PCR) and western blotting. **F** ChIP-Seq results were validated by qChIP analysis of the indicated genes. After DMSO or sorafenib (10 μM) treatment for 24 h, PLC/PRF/5 cells were collected, and qChIP experiments were performed with the indicated antibodies. **G** PLC/PRF/5 cells were co-transiently transfected with FOXQ1^WT^ or FOXQ1^S248A^ and the luciferase reporter gene driven by the ETHE1 promoter (ETHE1-Luc), the luciferase activity was detected after treatment with DMSO or sorafenib (10 μM) for 24 h. Relative luciferase activity was calculated by dividing firefly luciferase activity by renilla luciferase activity and is shown relative to controls. *, *P* < 0.05; **, *P* < 0.01; ***, *P* < 0.001.

To identify the downstream target genes of FOXQ1 regulating ferroptosis, we treated PLC/PRF/5 cells with DMSO or sorafenib and performed ChIP-Seq. The results showed that there were 311 differentially expressed genes in the DMSO group and 289 in the sorafenib group, of which 61 genes were at the intersection of the two groups (Fig. 6A). Next, we seek for potential target genes in the disjoint partial-area of sorafenib group. Due to the fact that ferroptosis is driven by abnormal metabolism, some metabolism related genes may play an important role in it, such as GLS2, NOX4 and ETHE1, were selected as potential downstream target genes of FOXQ1. The relationship between FOXQ1 and these genes was verified by qPCR, and the results showed that FOXQ1 increased the expression of ETHE1 after sorafenib treatment but had no obvious regulatory effect on GLS2 and NOX4 (Fig. 6B). The GSH antioxidant system and iron metabolism have been reported to play a central role in the regulation of ferroptosis. ETHE1 is a persulfide dioxygenase that generates GSH and binds free iron [26, 27], which may inhibit ferroptosis. Through western blot and qPCR verification, it was found that FOXQ1 could increase ETHE1 expression at protein level and mRNA level after sorafenib treatment but had no obvious effect on ETHE1 in the DMSO treatment group. Furthermore, such promoting effect of FOXQ1 on ETHE1 can be eliminated with the FOXQ1 serine 248 site mutation (Fig. 6C–E). The results of ChIP‒qPCR showed that FOXQ1 was much more significantly combined with the promoter region of ETHE1 after sorafenib treatment (Fig. 6F). The results of the dual luciferase reporter assay showed that overexpression of wild-type FOXQ1 rather than FOXQ1 with S248A mutation increased the transcriptional activity of ETHE1 after sorafenib treatment (Fig. 6G). These results suggest that serine 248 site phosphorylation-driven FOXQ1 can target and activate the transcriptional activity of ETHE1.

### ETHE1 is a target gene of FOXQ1 that regulates ferroptosis in HCC

**Fig. 7:**
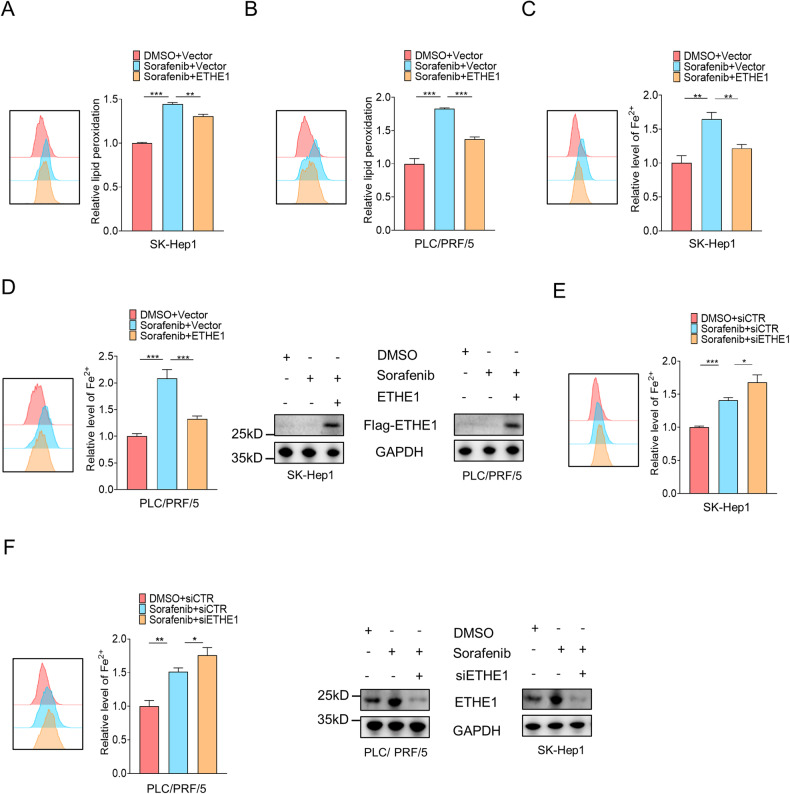
ETHE1 is a target gene of FOXQ1 regulating ferroptosis in HCC. SK-Hep1 and PLC/PRF/5 cells were transiently transfected with Vector and ETHE1, then the lipid peroxidation (**A**, **B**) and iron content (**C**, **D**) were assessed by flow cytometry after DMSO and sorafenib (10 μM) treatment. **E**, **F** Effect of ETHE1 knockdown on iron content in SK-Hep1 cells and PLC/PRF/5. *P* values were determined by Student’s *t*-test. *, *P* < 0.05; **, *P* < 0.01; ***, *P* < 0.001.

**Fig. 8:**
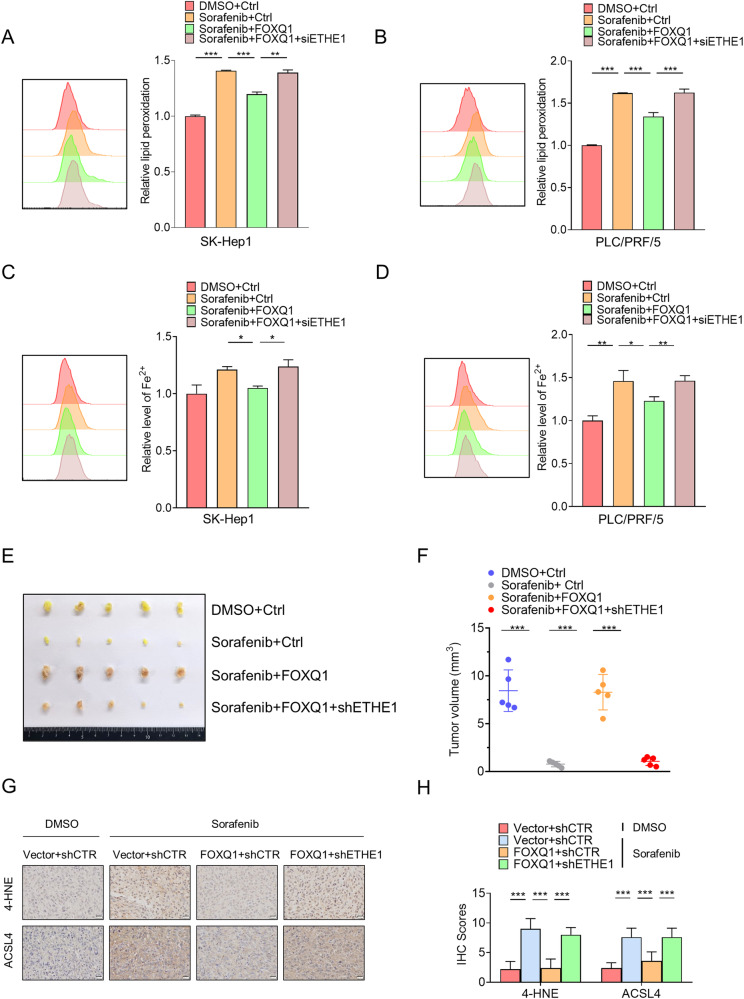
FOXQ1 regulates ferroptosis in HCC by targeting and activating ETHE1. SK-Hep1 and PLC/PRF/5 cells were transiently transfected with FLAG-FOXQ1 or/and siETHE1, then the lipid peroxidation (**A**, **B**) and iron content (**C**, **D**) were assessed by flow cytometry after DMSO and sorafenib (10 μM) treatment. **E**, **F** SK-Hep1 cells were infected with lentiviruses carrying vector+shCtrl (pLenti-CMV-GFP+pLKO.1), FOXQ1+shCtrl (pLenti-CMV-GFP-FOXQ1+pLKO.1), FOXQ1+shETHE1(pLenti-CMV-GFP-FOXQ1+pLKO.1-shETHE1). HCC cells were injected into nude mice via the left posterior flanks of 7-week-old immunodeficient female nude mice. The mice were divided into groups and injected intraperitoneally with sorafenib (30 mg/kg/d) for 2 weeks (*n* = 5 for each group). Tumors were collected and photographed (**E**) and tumor volume was measured (**F**). **G**, **H** Immunohistochemical staining for 4-HNE and ACSL4 in subcutaneous tumors of indicated mice. Scale bar= 20 μm. *P* values were determined by Student’s *t* test. *, *P* < 0.05; **, *P* < 0.01; ***, *P* < 0.001.

It has been reported that ETHE1 contains conserved amino acid residues for iron ion binding [28] (Fig. S1C), so we speculate that ETHE1 may be involved in the regulation of ferroptosis by binding ions to reduce intracellular free iron ions. Our results showed that overexpression of ETHE1 suppressed the elevated lipid peroxidation and ions levels induced by sorafenib, while knockdown of ETHE1 could increase ions levels (Fig. 7A–F). Furthermore, knockdown of ETHE1 rescued the decreased intracellular lipid peroxidation and iron ion levels caused by overexpression of FOXQ1 (Fig. 8A–D). The transfection efficiency of each transfection group is shown in Fig. S4A.

To determine whether FOXQ1 inhibits the anticancer activity of sorafenib in vivo through ETHE1, SK-Hep1 cells stably expressing FOXQ1 and stably expressing FOXQ1 and shETHE1 were transplanted subcutaneously into nude mice. Lentivirus transfection efficiency is shown in Figure S4B. Seven days later, the mice began to receive sorafenib treatment for 12 days. Compared with the control group, the tumor volume was significantly decreased after sorafenib treatment (Fig. 8E, F), and the expression of ACSL4 and 4HNE were enhanced. Overexpression of FOXQ1 significantly increased the volume of the tumors, and the expression of ACSL4 and 4HNE was decreased. However, knockdown of ETHE1 reversed the effect of FOXQ1 (Fig. 8G, H). These results suggest that FOXQ1 inhibits sorafenib-induced ferroptosis by targeting and activating ETHE1.

## Discussion

**Fig. 9:**
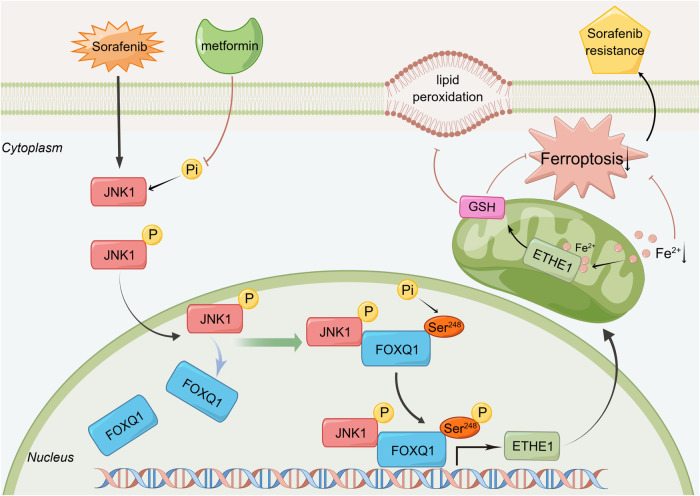
Schematic diagram of molecular mechanism of FOXQ1 regulating ferroptosis in HCC. Sorafenib phosphorylates JNK1, and the phosphorylated JNK1 enters the nucleus and binds to FOXQ1 to further activate the phosphorylation at serine 248 site of FOXQ1. Then the phosphorylated FOXQ1 binds to the promoter region of ETHE1 and transcriptionally activates ETHE1 to inhibit ferroptosis.

Despite advances in HCC treatment over the past decade, the efficacy of currently available therapies remains unsatisfactory. Sorafenib-induced ferroptosis has great potential in the treatment of HCC. However, drug resistance is an important issue limiting its therapeutic efficacy. In this study, we constructed sorafenib-resistant cell lines and found that FOXQ1 was significantly highly expressed in the drug-resistant lines. Mechanistically, FOXQ1 is phosphorylated by JNK1, the phosphokinase functionally activated by sorafenib. FOXQ1 phosphorylation, then, promotes the expression of ETHE1, thereby inhibiting sorafenib-induced ferroptosis in HCC (Fig. 9). Therefore, our study provides two therapeutic strategies to enhance sorafenib-induced ferroptosis: one is to target JNK1, an upstream kinase of FOXQ1 phosphorylation, to reduce the regulation of ferroptosis by inhibiting the phosphorylation of FOXQ1, and the other is to target FOXQ1 and its downstream signaling pathways. This study has identified new therapeutic approaches to overcome sorafenib resistance by regulating the FOXQ1/ETHE1 axis.

FOXQ1 functions as a transcription factor in tumors and regulates multiple stages of tumor development, including the tumor invasion and metastasis, cell proliferation and angiogenesis [10, 29, 30]. Several regulatory mechanisms of FOXQ1 in normal and tumor cells have been reported. It has been reported that noncoding RNAs such as miR-124, miR-422a, and miR-1271 can promote tumor proliferation, migration and invasion by regulating the expression of FOXQ1 [31–33]. FOXQ1 in cancer cells can also be activated through oncogenic signaling pathways. The main pathways involved are the Wnt/β-catenin signaling and transforming growth factor (TGF)-β signaling pathways. Christensen et al. reported that as the downstream intermediary of the Wnt/β-catenin signaling pathway, FOXQ1 can be regulated through the recognized transcription factor-4 binding site [34]. Likewise, TGF-β treatment was reported to significantly increased FOXQ1 mRNA and protein levels [35]. In addition, the expression of FOXQ1 may also be regulated by transcriptional coregulators. NAC1 is a coregulatory transcriptional factor involved in various biological processes [36–38]. Gao et al. reported that the expression of FOXQ1 can be transcriptionally regulated by NAC1 [39]. In this study, although FOXQ1 expression was not significantly changed under short-term sorafenib stimulation, it was significantly increased in cells with long-term stimulation and tolerance to sorafenib, which affected the sensitivity of hepatocellular carcinoma cells to sorafenib. At present, how long-term use of sorafenib increases the expression level of FOXQ1 is still unknown. Further in-depth research may focus on the mechanism of the elevated expression of FOXQ1 in sorafenib-resistant cells to provide a new solution for overcoming sorafenib resistance.

Ferroptosis has been recognized to play an important role in chemotherapy, radiotherapy, immunotherapy, and targeted therapy for many cancers [40–42]. The unique metabolism of cancer cells makes them inherently susceptible to ferroptosis. Thus, the use of ferroptosis inducers offers a practical approach to cancer therapy [43, 44]. Sorafenib, as an inducer of ferroptosis, plays an important role in the treatment of HCC [45]. Sorafenib has been shown to induces ferroptosis by inhibiting the system Xc(-). Treatment of HT-1080 cells with various concentrations of sorafenib was studied to cause a concentration-dependent inhibition of system Xc(-) function(5). Atf4-dependent tumor promotion was shown to be mediated by transcriptional targeting system Xc(-), whereas ATF4-induced proliferation could be attenuated by sorafenib(45). In this study, we found that after sorafenib treatment, FOXQ1 suppressed ferroptosis markers in HCC cells. ChIP-seq analysis indicated that ETHE1 is a potential target of FOXQ1. ETHE1 is a persulfide dioxygenase that converts GSSH to GSH in mitochondria [27, 46]. GSH is an important reducing agent in cells that can inhibit ferroptosis through the antioxidant pathway of GPX4 [47]. It has also been reported that ETHE1 needs to bind intracellular free iron to exert its enzymatic activity [28, 48], which may result in the reduction of intracellular free iron and inhibit ferroptosis. Our experimental results show that ETHE1 can significantly inhibit lipid peroxidation and iron levels in HCC cells, indicating that ETHE1 is an effective inhibitor of ferroptosis in HCC.

Meanwhile, the target genes and motifs of FOXQ1 were significantly altered after sorafenib treatment (Figure S2 A). It has been reported that some proteins will undergo structural changes after posttranslational modifications, such as succinylation at K311 of PKM2, which promotes the transformation of its tetramers to dimers and changes its cellular localization and function [49]. YY1 was shown to have dimeric forms, which play different transcriptional roles compared to the monomeric form [50]. We speculate that FOXQ1 also undergoes protein structure changes after phosphorylation to exercise different transcription modes. A native polyacrylamide gel electrophoresis experiment (native-PAGE) was conducted to prove our conjecture and the result showed that after sorafenib treatment, FOXQ1 had a tendency to change from monomerization to multimerization, and it returned to monomerization after mutation of serine 248 (Fig. S2B–E). This indicates that the serine 248 site phosphorylated FOXQ1 leads to multimerization and may be part of the reason why the target genes and motifs of FOXQ1 are greatly changed after sorafenib treatment.

Since phosphorylation plays an important role in the mechanism by which FOXQ1 regulates ferroptosis, targeting the phosphorylated upstream kinase JNK1 of FOXQ1 will be a promising solution to rescue sorafenib resistance caused by FOXQ1. Many JNK inhibitors have been discovered thus far; unfortunately, none of them have been approved for use in humans, and only a few have entered clinical trials (such as AS602801, CC-930, AGI-1067, CC-401) (51). This is mainly because JNK has several highly homologous isoforms functioning in different pathogenic processes, while the current JNK inhibitors lack specificity for specific JNK isoforms [51, 52]. Such indiscriminate targeting results in broad inhibition of phosphorylation of JNK substrates and may lead to cytotoxicity. In our study, the phosphorylation of JNK was activated by sorafenib, so future research should focus on exploring the safety and efficacy of the combined use of sorafenib and JNK inhibitors in HCC. Metformin, a derivative of biguanide, has been used for nearly a century due to its safety and low cost [53]. A large number of studies have proven that metformin can inhibit the phosphorylation of JNK [54, 55], and some studies have explored the effect of sorafenib combined with metformin [56, 57]. Interestingly, our study found that metformin can inhibit the phosphorylation of JNK (45 kDa) induced by sorafenib and inhibit the phosphorylation of FOXQ1 (Fig. S3 A-D). More importantly, the lipid peroxidation and iron levels suppressed by overexpression of FOXQ1 were rescued by metformin treatment (Fig. S3E–H). Hence, metformin may be an effective way to rescue patients with sorafenib resistance due to the high expression of FOXQ1. However, combining metformin and sorafenib is still controversial [58], possibly due to the lack of effective biomarkers for patient selection. Our study shows that FOXQ1 may be an effective biomarker for sorafenib combination therapy, which requires further in-depth exploration to develop personalized therapy.

This study conducted a detailed and novel mechanistic study and elucidated how FOXQ1 is involved in sensitivity to sorafenib treatment. We found that phosphorylation of FOXQ1 by JNK1 transcriptionally activates ETHE1, enhancing HCC resistance to sorafenib-induced ferroptosis. Considering the non-negligible role of FOXQ1 in sensitivity to sorafenib therapy, targeting the JNK1-FOXQ1-ETHE1 axis may provide avenues to create novel biomarkers and therapeutics to overcome sorafenib resistance.

### Supplementary information

Supplementary Materials-all

Original western blots

## Data Availability

Data and materials supporting the findings of this study are available from the corresponding author upon reasonable request.

## Material availability

Data and materials supporting the findings of this study are available from the corresponding author upon reasonable request.
