# Supplementary material for: Phosphorylated FOXQ1, a novel substrate of JNK1, inhibits sorafenib-induced ferroptosis by activating ETHE1 in hepatocellular carcinoma
Source: Cell Death Dis. 2024 Jun 5;15(6):395. doi: 10.1038/s41419-024-06789-1 (PMC11153576; doi:10.1038/s41419-024-06789-1)
Supplement: Supplementary file 1 — Supplementary Materials-all [file 41419_2024_6789_MOESM1_ESM.pdf]

Figure S1

A

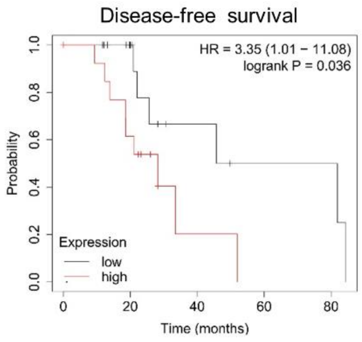

B

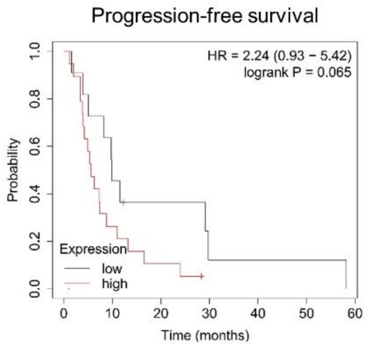

C

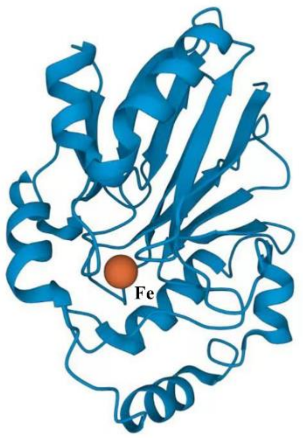

Figure S1. **A, B** The relationship between FOXQ1 expression and prognosis in 30 sorafenib users. (A: Disease-free survival, B: Progression-free survival). **C** Schematic diagram of the protein structure of ETHE1.

Figure S2

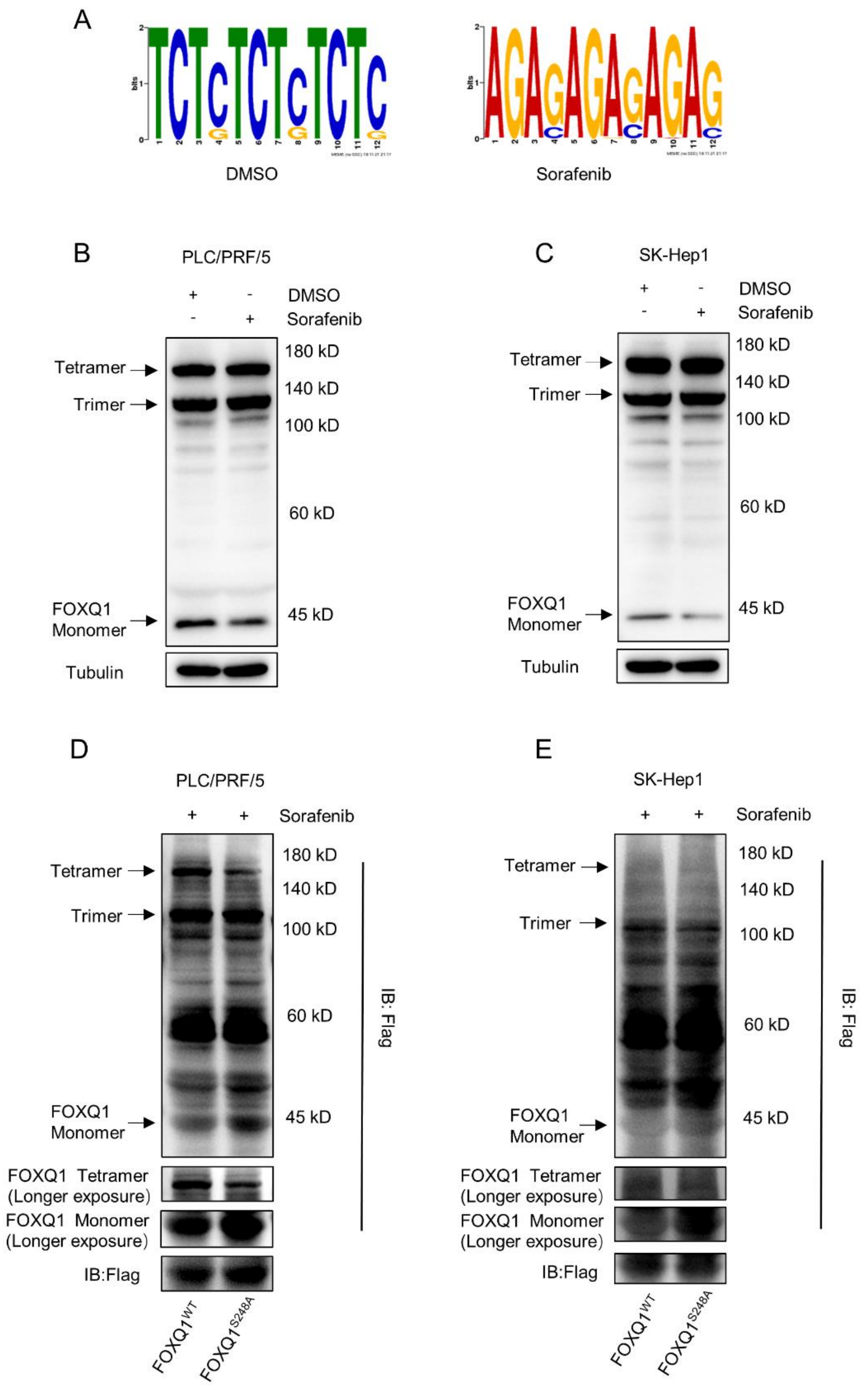

Figure S2. **A** The motif of FOXQ1 in DMSO group and Sorafenib group. **B, C** The changes of FOXQ1 monomer, trimer and tetramer after sorafenib treatment were verified by native polyacrylamide gel electrophoresis experiment (native-PAGE). **D, E** The changes of FOXQ1 monomer, trimer and tetramer after the FOXQ1 serine 248 site mutation were verified by native-PAGE.

Figure S3

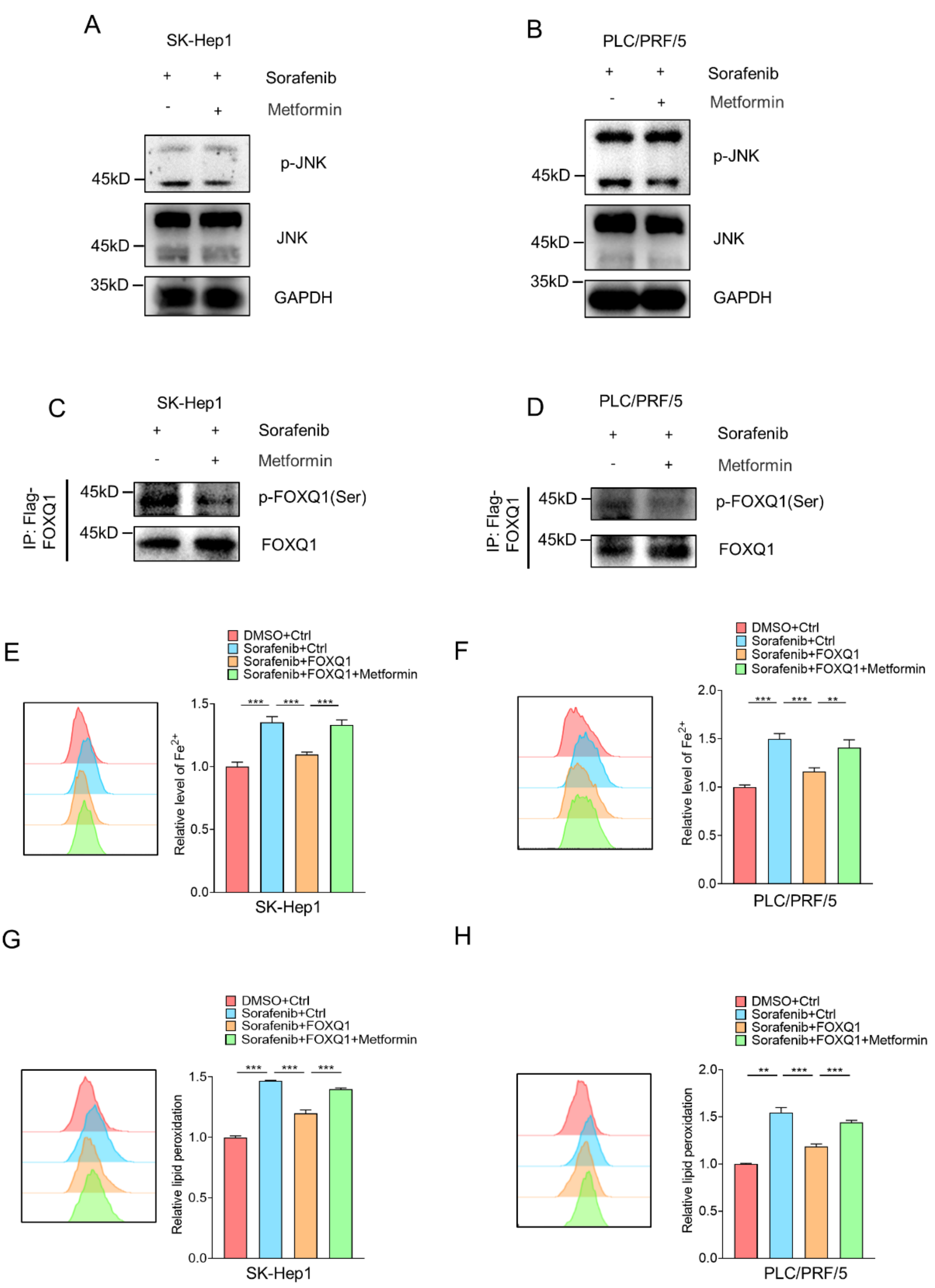

Figure S3. **A, B** Effect of Metformin on JNK phosphorylation. **C, D** Effect of Metformin on phosphorylation of FOXQ1 at serine. **E-H** Effect of Metformin expression on FOXQ1-regulated cellular iron content and lipid peroxidation.

Figure S4

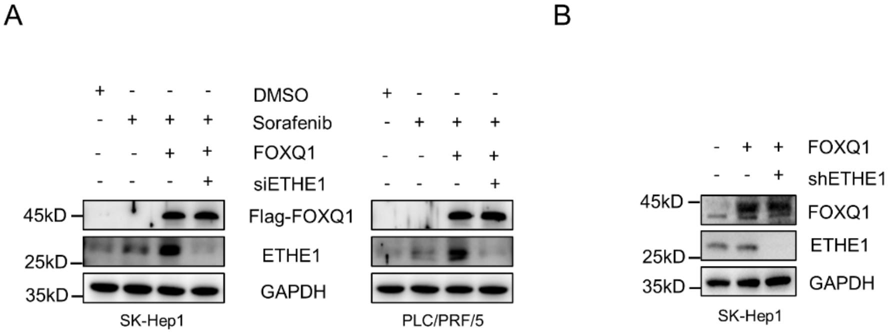

Figure S4. **A** The transfection efficiency of each transfection group of Figure 8A-D. **B** Lentivirus transfection efficiency of Figure 8E-H.

Table S1. Sequence of shRNA

	Sense
shCtrl	5’-TTCTCCGAACGTGTCACGT-3’
shETHE1	5’-CAGCAGATAGACTTTGCTGTT-3’

Table S2. Sequence of siRNA

	Sense	Anti-sense
siETHE1-NC	UUCUCCGAACGUGUCACGUTT	ACGUGACACGUUCGGAGAATT
siETHE1	GAUCUACCCUGCUCACGAUTT	AUCGUGAGCAGGGUAGAUCTT
siFOXQ1-NC	UUCUCCGAACGUGUCACGUTT	ACGUGACACGUUCGGAGAATT
siFOXQ1-#1	CCAUUGAUUUAUGUCCCUUTT	AAGGGACAUAAAUCAAUGGTT
siFOXQ1-#2	GCAACGGGCUACAGCUUUATT	UAAAGCUGUAGCCCGUUGCTT

Table S3. Sequence of The PCR primers

	Forward（5’-3’）	Reverse（3’-5’）
NOX4	GCCAGAGTATCACTACCTCCAC	CTCGGAGGTAAGCCAAGAGTGT
GLS2	TGAGGCACTGTGCTCGGAAGTT	TCGAAGAGCTGAGACATCGCCA
ETHE1	CTTCGTCCTGAATGACCACAGC	CAGACAGTCTCCTGGAAGTGTG
FOXD1	GATCTGTGAGTTCATCAGCGGC	TGACGAAGCAGTCGTTGAGCGA
FOXM1	TCTGCCAATGGCAAGGTCTCCT	CTGGATTCGGTCGTTTCTGCTG
FOXQ1	CCTACTCGTACATCGCGCTCAT	TCGTTGAGCGAAAGGTTGTGGC
GAPDH	CAAGGTCATCCATGACAACTTTG	GTCCACCACCCTGTTGCTGTAG
