# Supplementary material for: Phosphorylated FOXQ1, a novel substrate of JNK1, inhibits sorafenib-induced ferroptosis by activating ETHE1 in hepatocellular carcinoma
Source: Cell Death Dis. 2024 Jun 5;15(6):395. doi: 10.1038/s41419-024-06789-1 (PMC11153576; doi:10.1038/s41419-024-06789-1)

Figure 2B

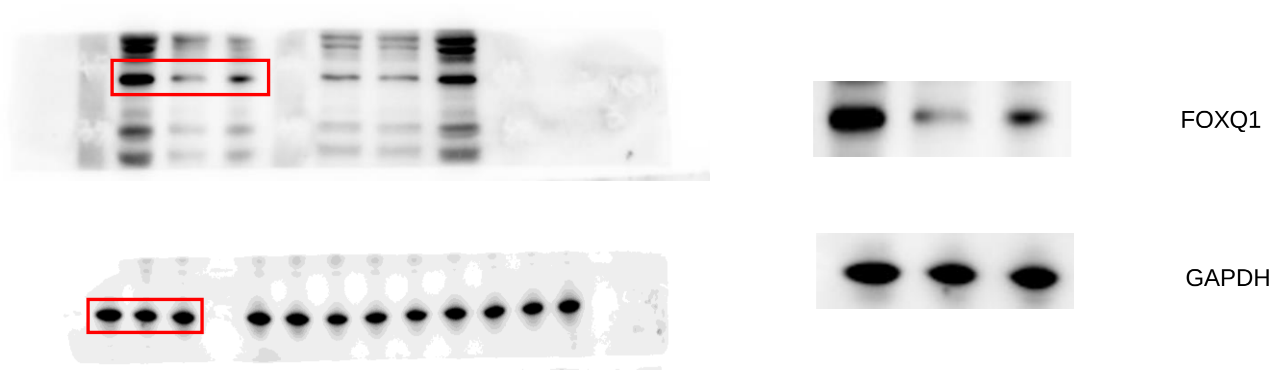

Figure 2E

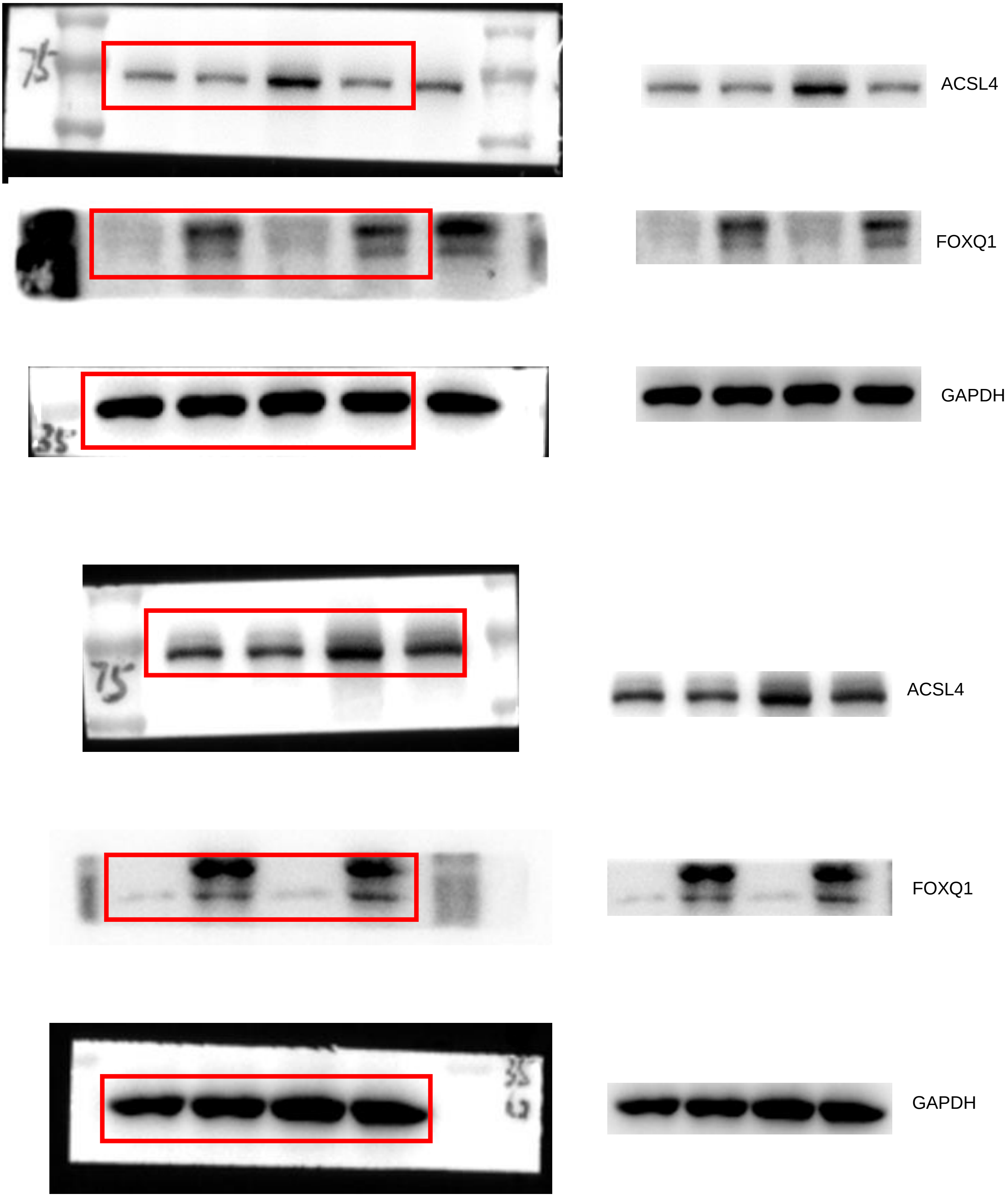

Figure 3A SK-Hep1

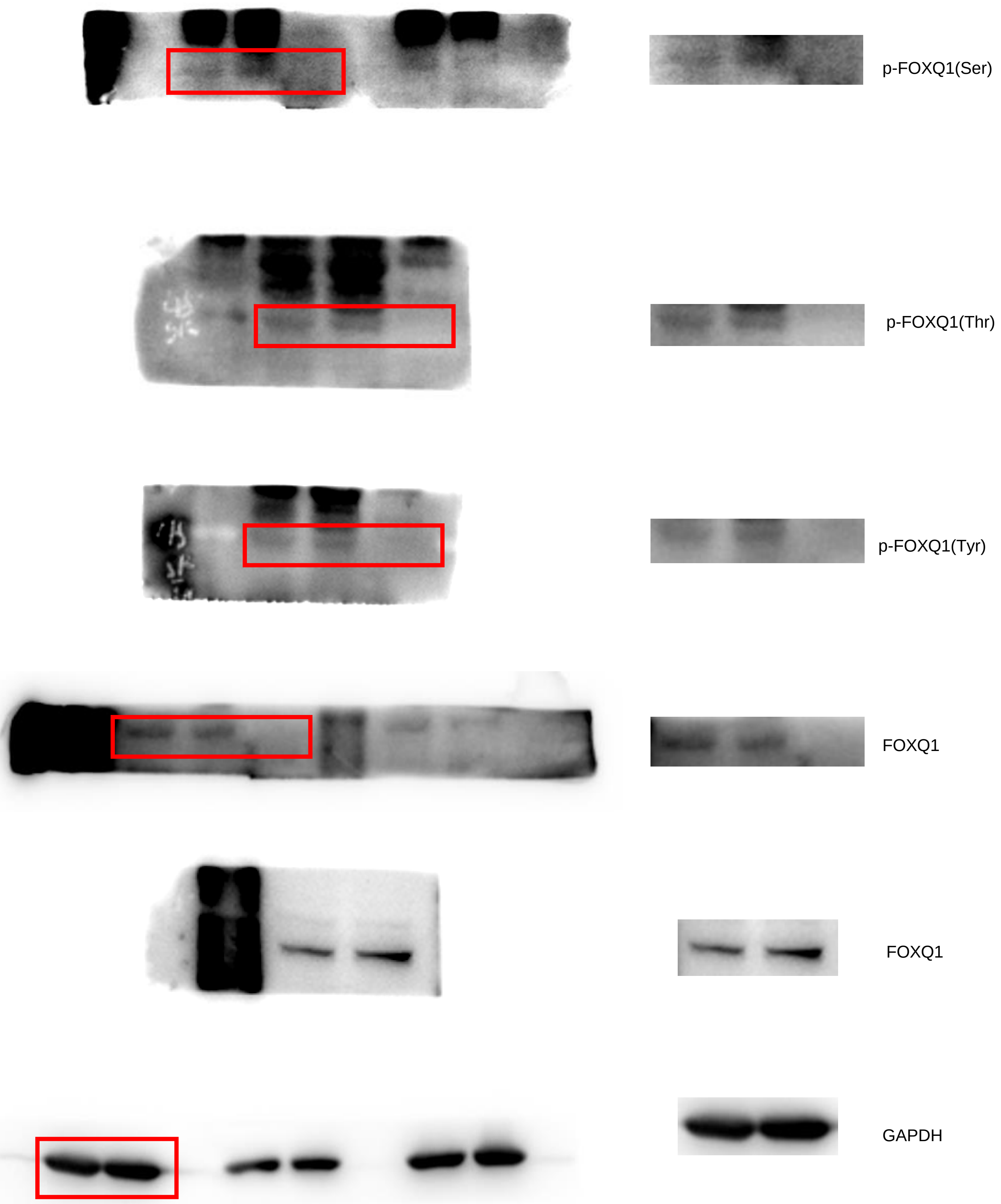

Figure 3A

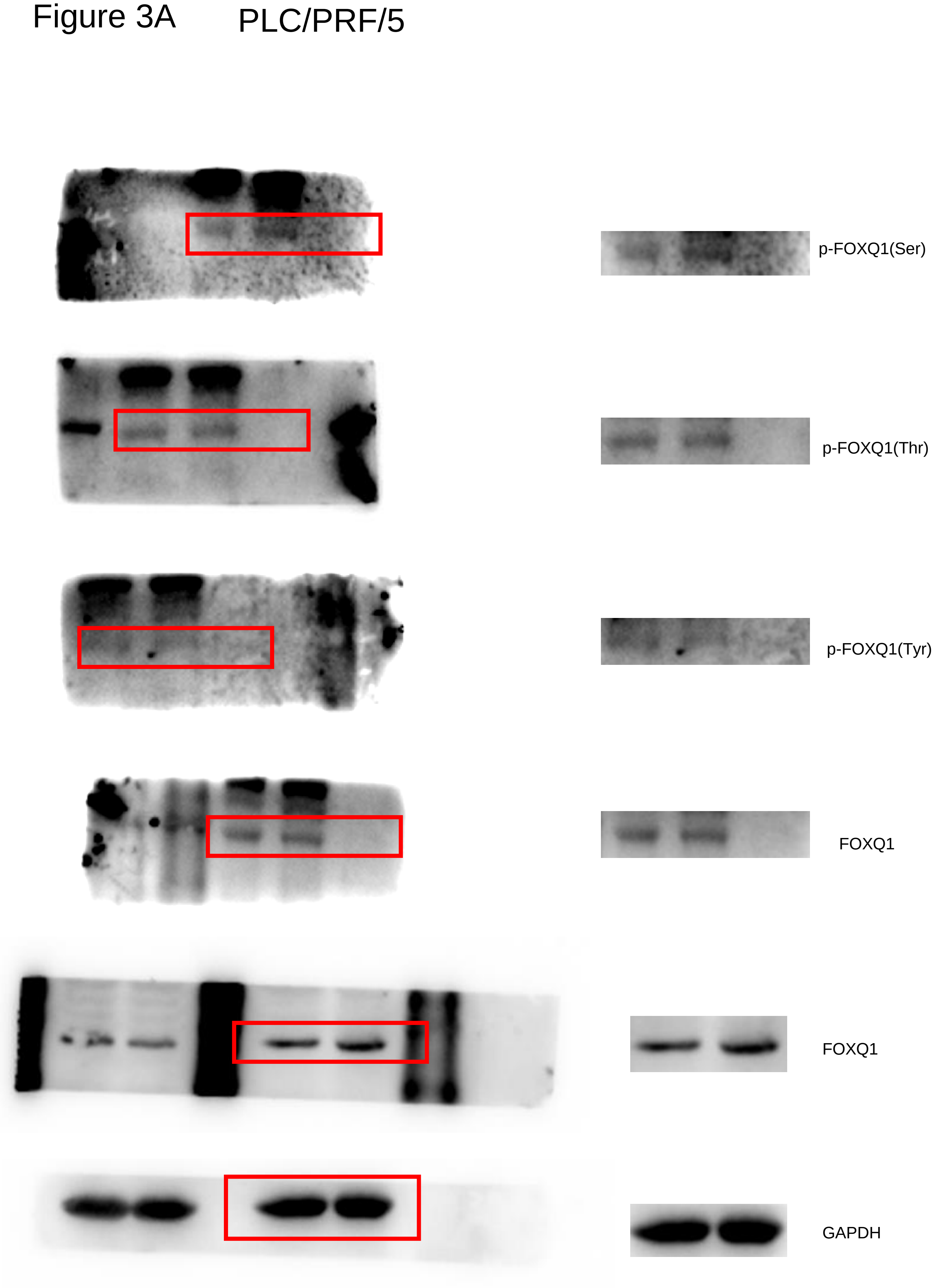

Figure 3B SK-Hep1

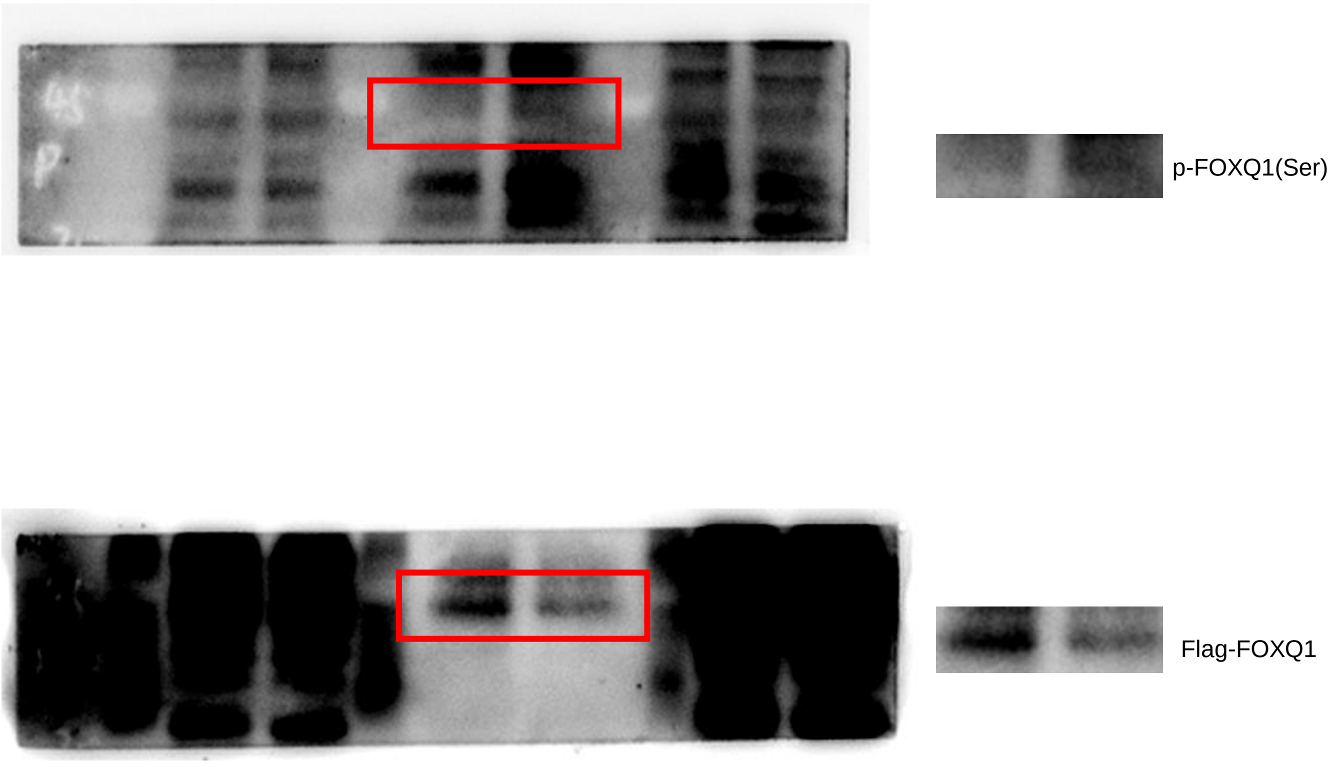

Figure 3B PLC/PRF/5

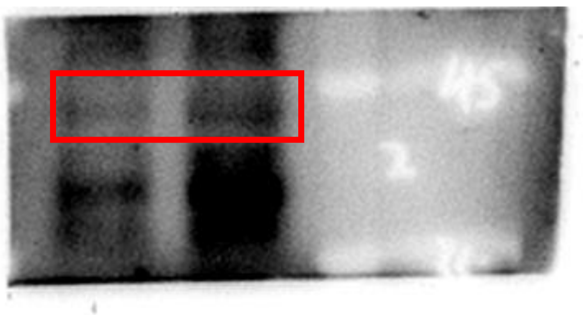

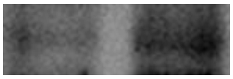

p-FOXQ1(Ser)

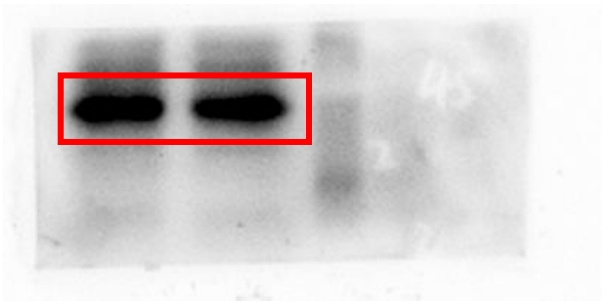

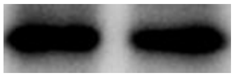

Flag-FOXQ1

Figure 3C SK-Hep1

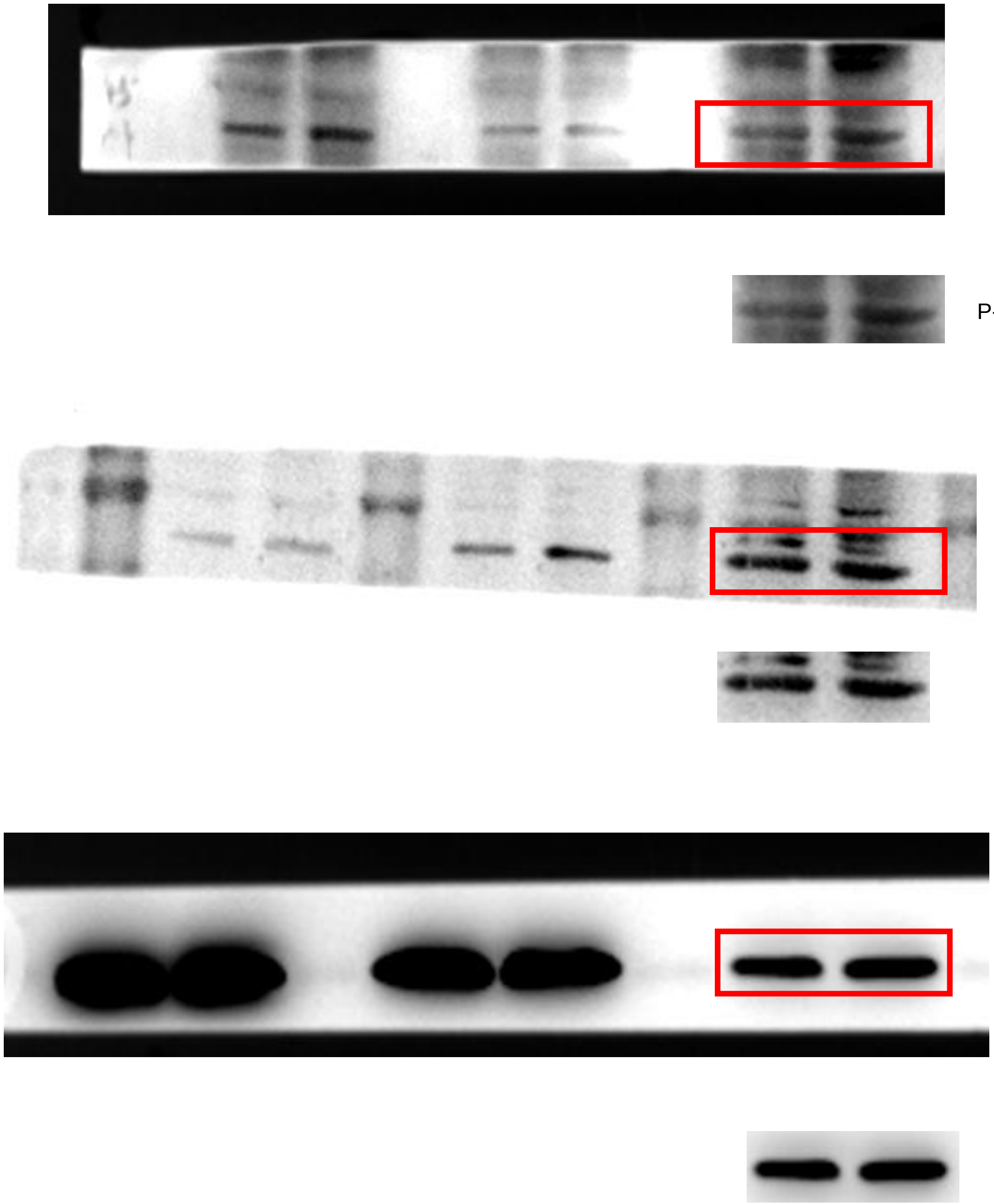

P-FOXQ1(S248)

FOXQ1

GAPDH

Figure 3C PLC/PRF/5

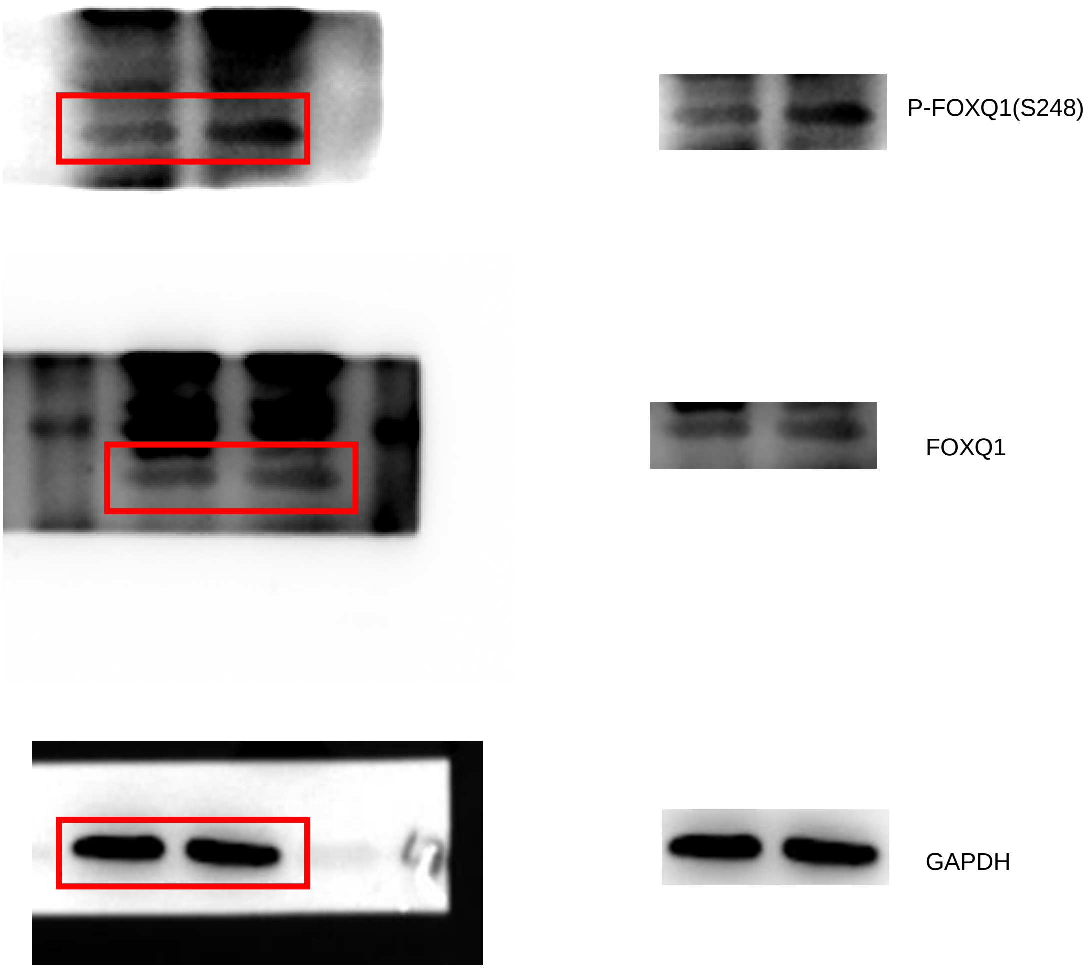

Figure 3D

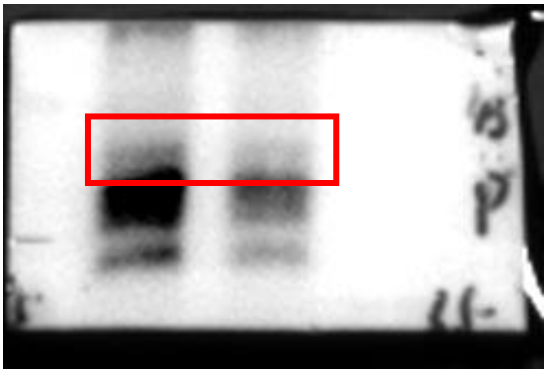

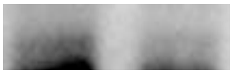

p-FOXQ1(Ser)

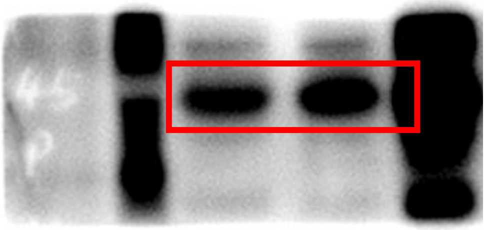

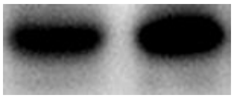

FOXQ1

Figure 4B SK-Hep1

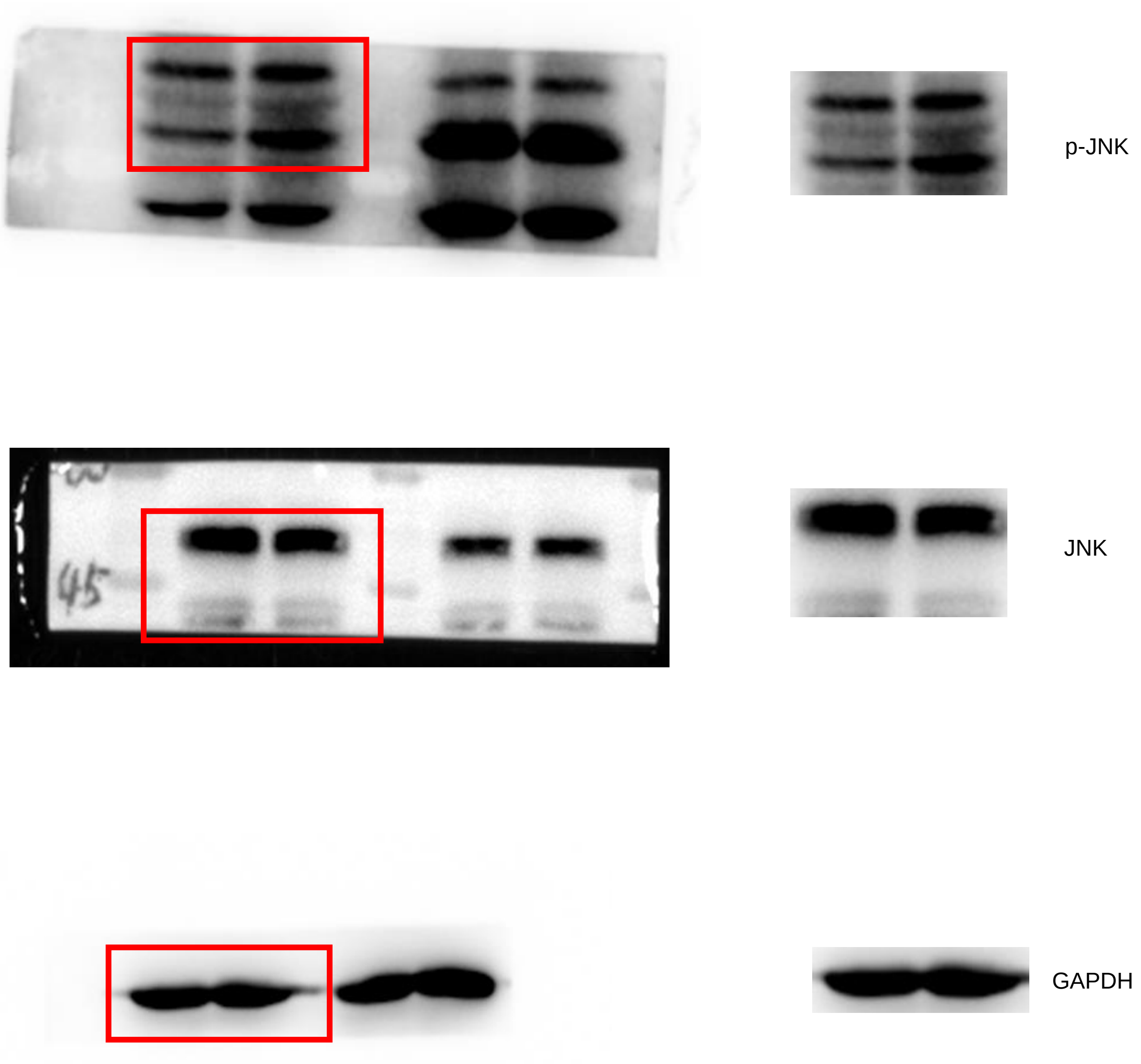

Figure 4B PLC/PRF/5

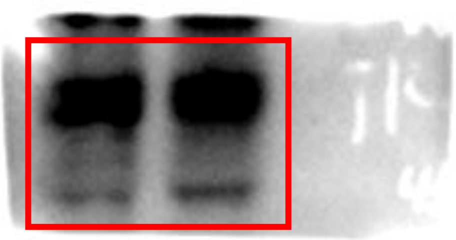

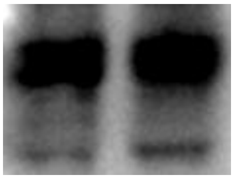

p-JNK

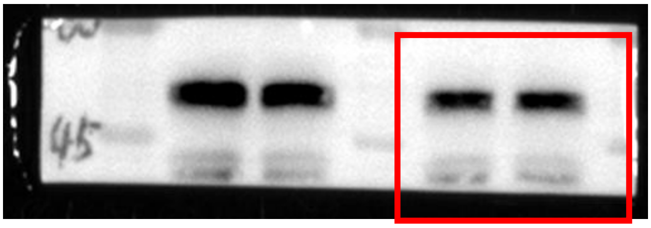

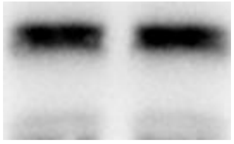

JNK

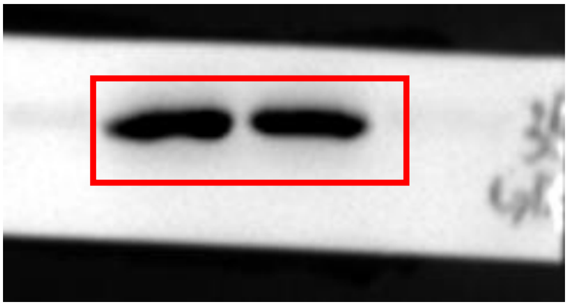

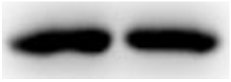

GAPDH

Figure 4C

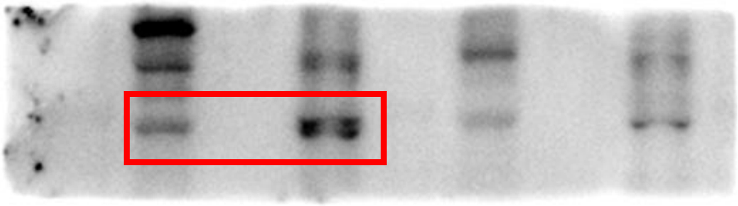

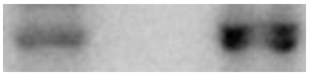

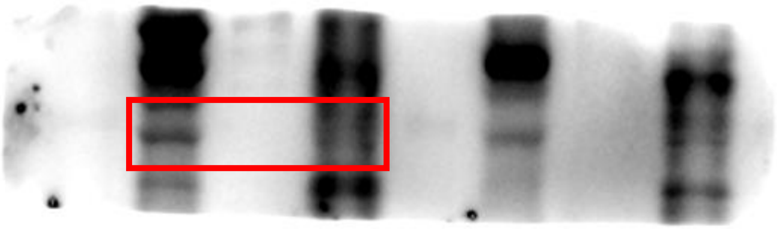

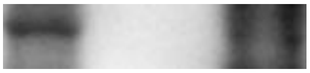

Figure 4D

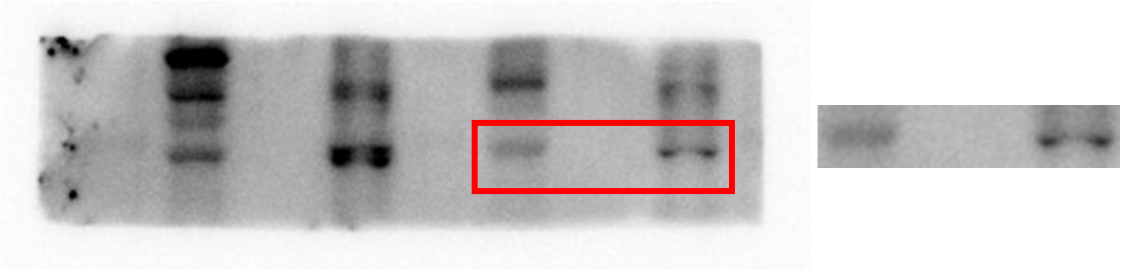

JNK1

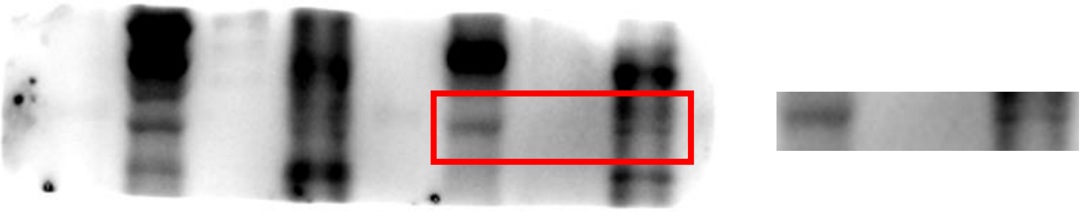

FOXQ1

Figure 4E SK-Hep1

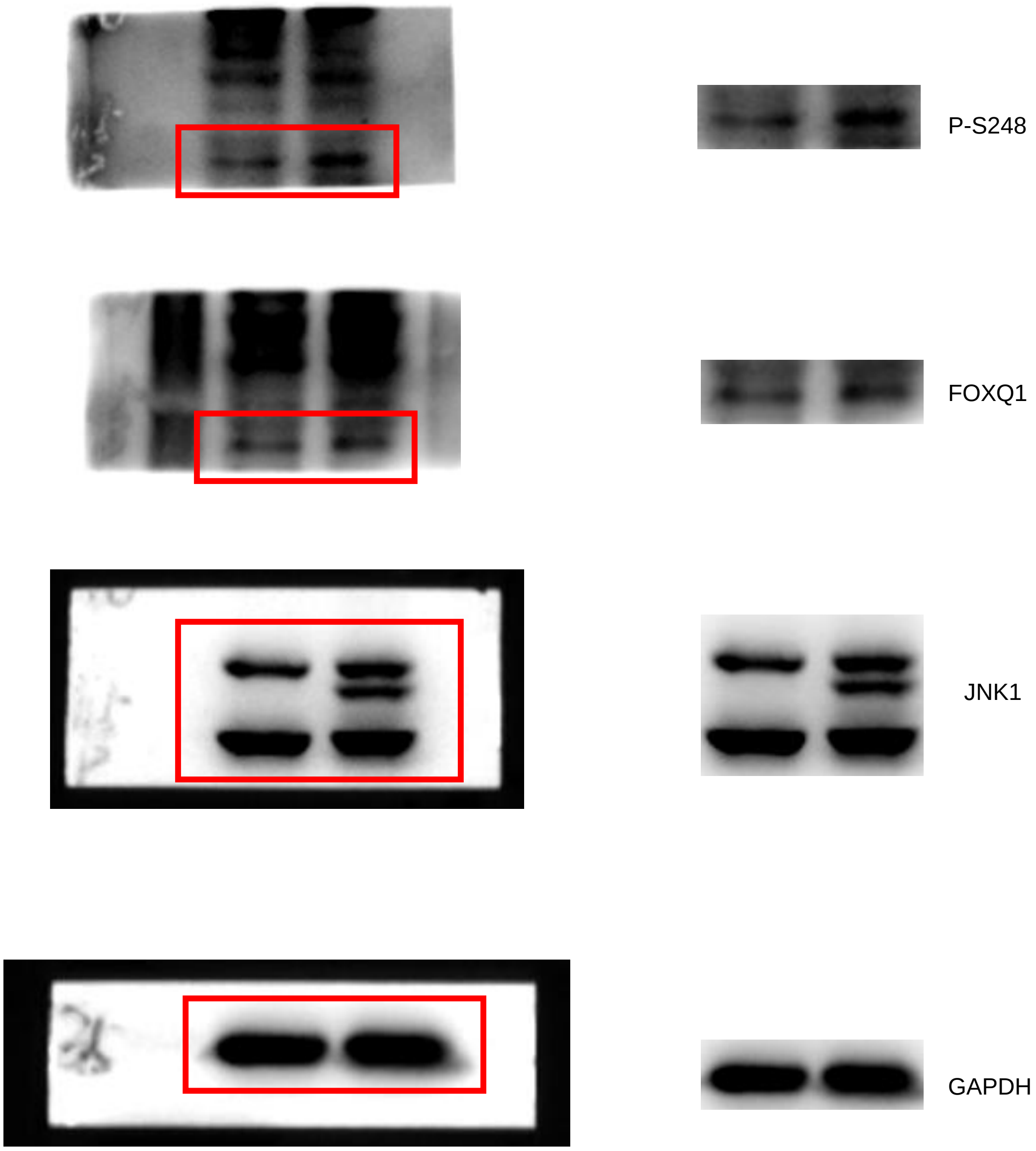

Figure 4E PLC/PRF/5

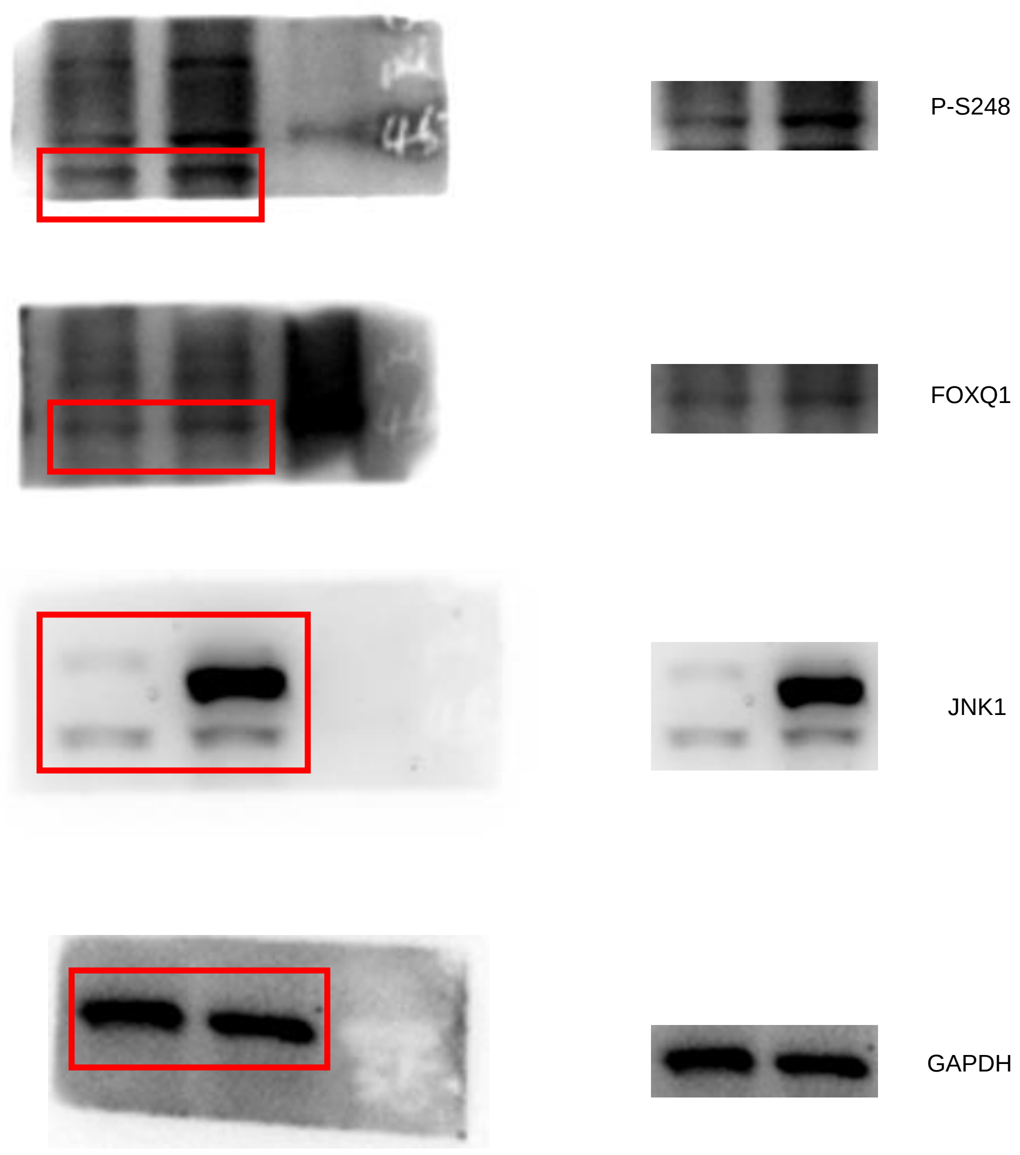

Figure 4F

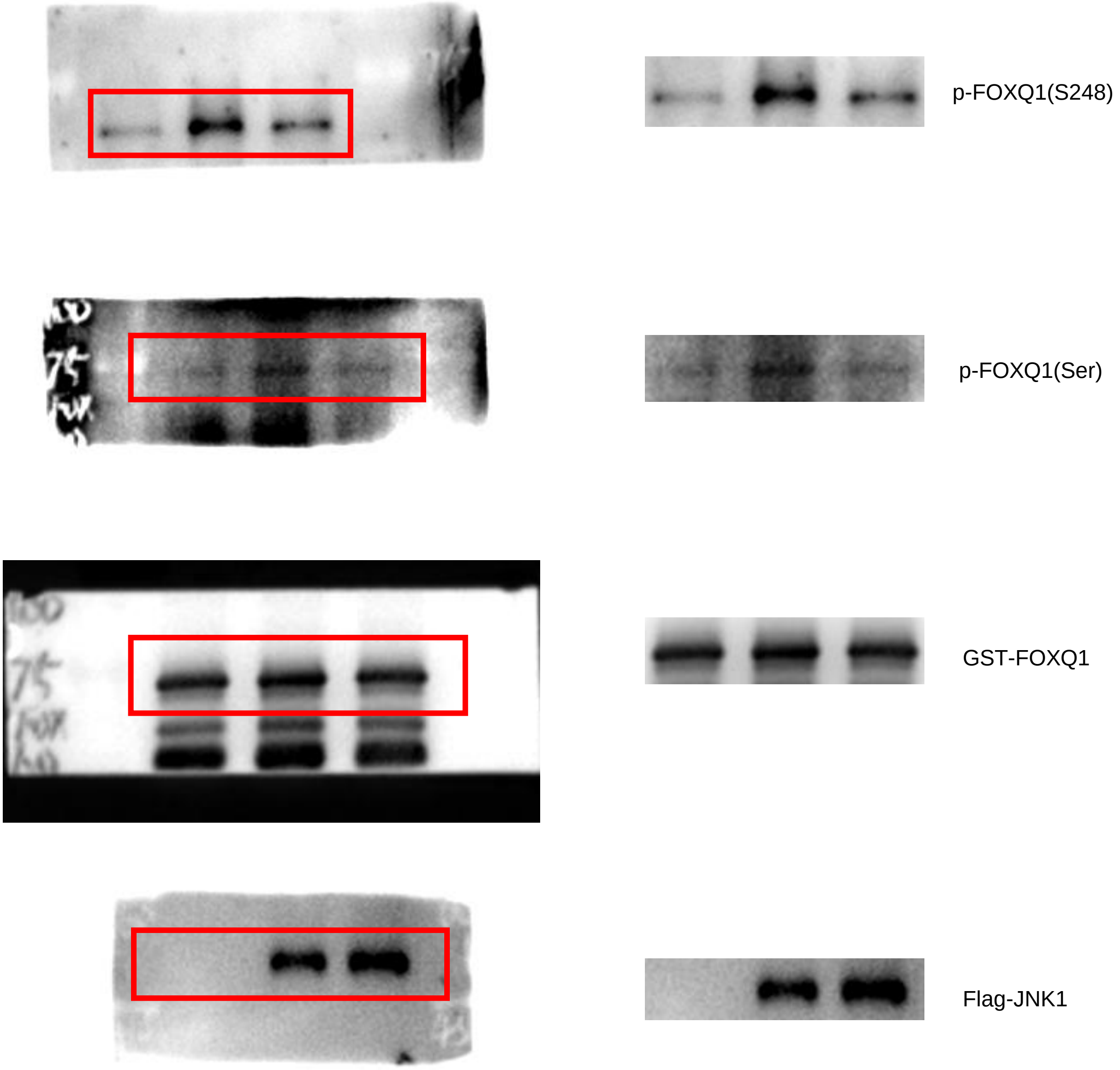

Figure 5A

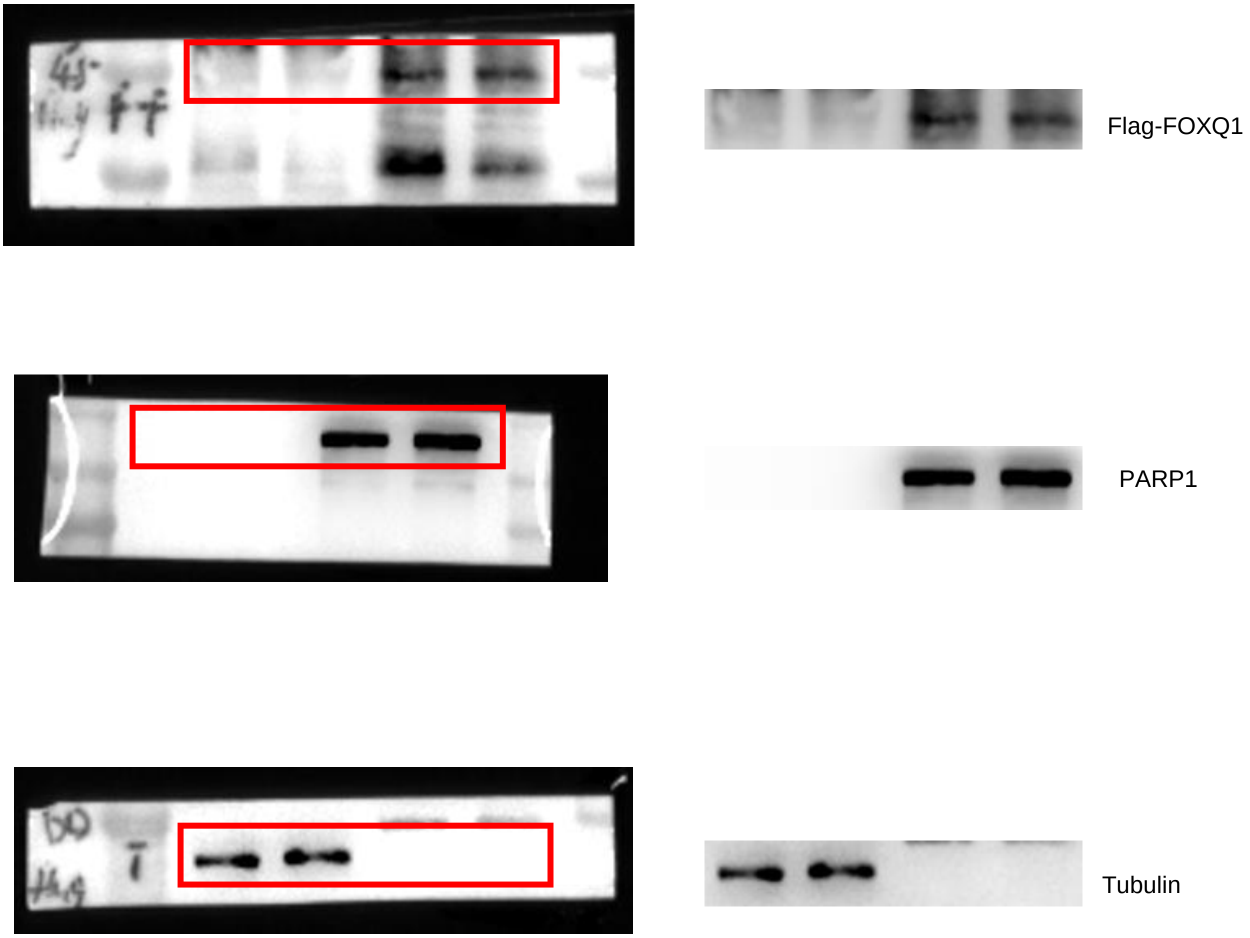

Figure 5B

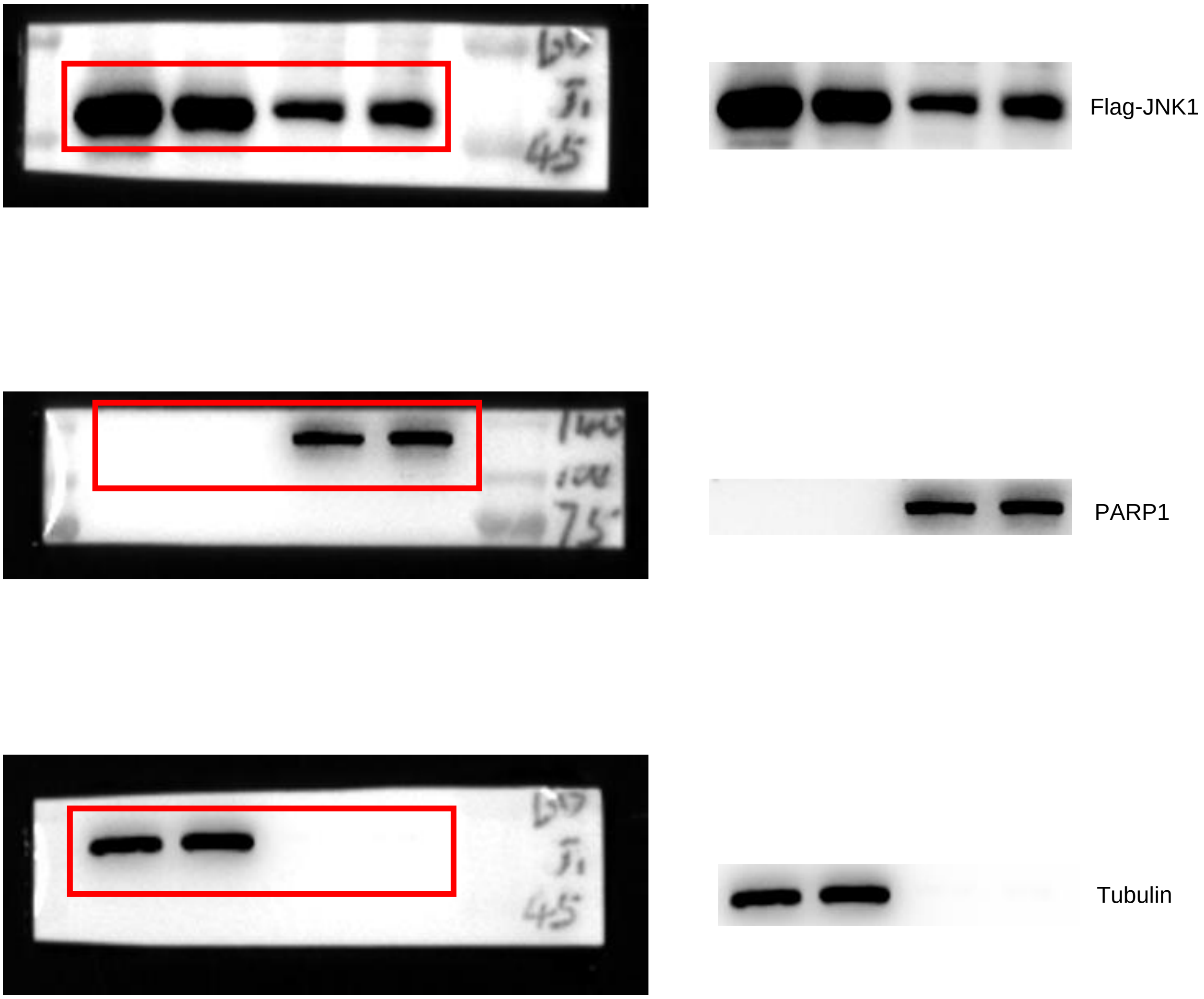

Figure 5C

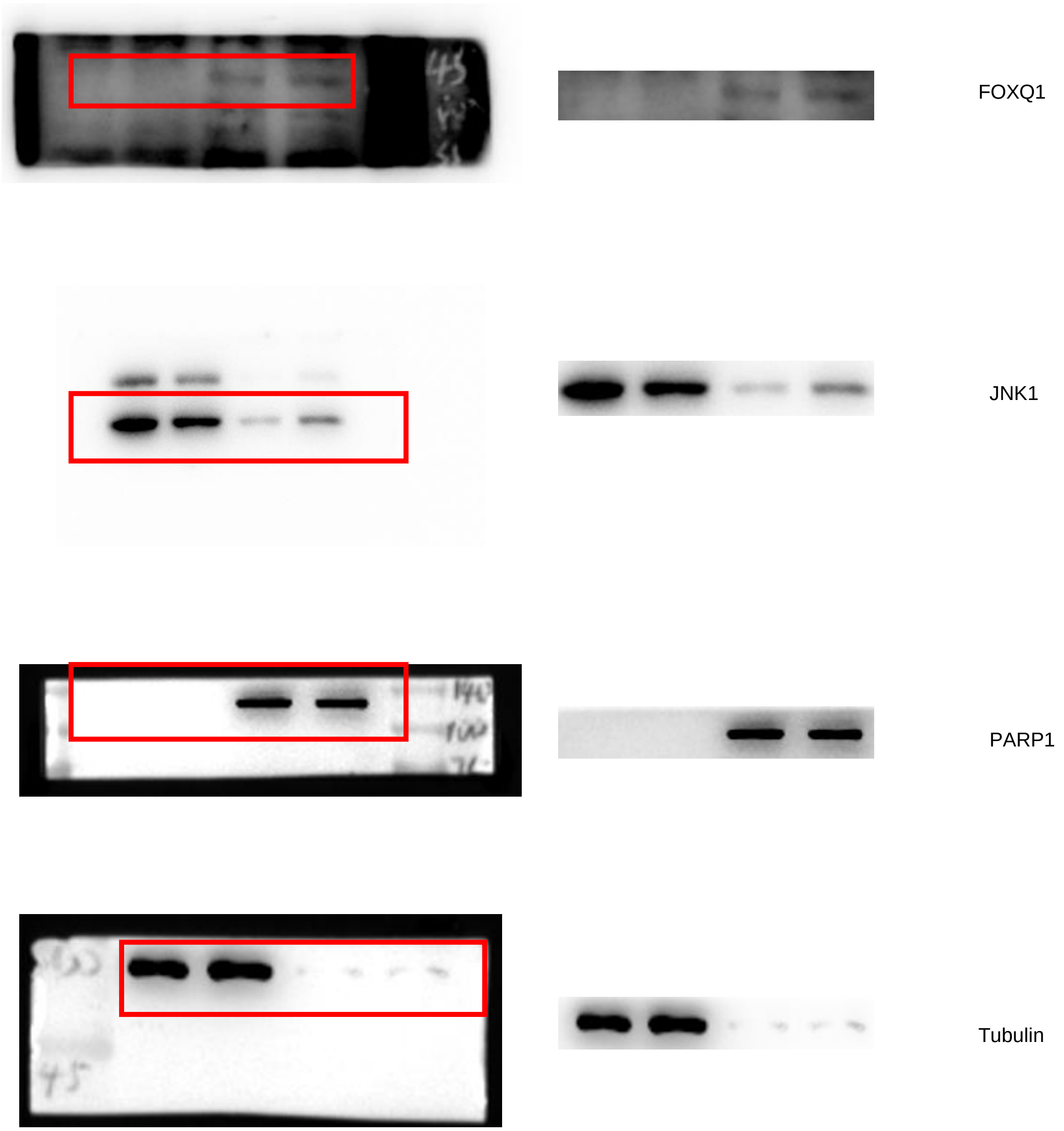

Figure 5D

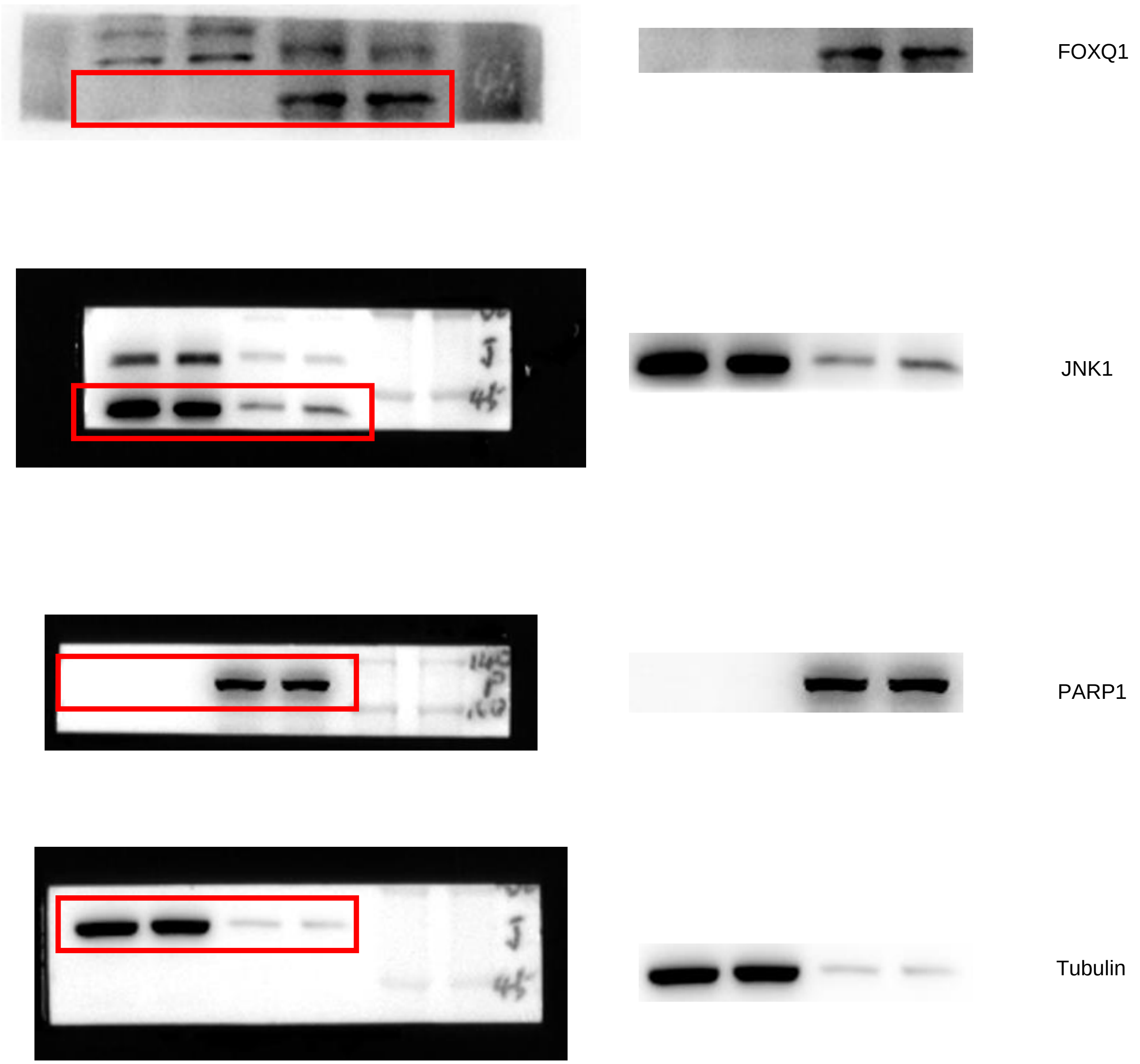

Figure 5E SK-Hep1

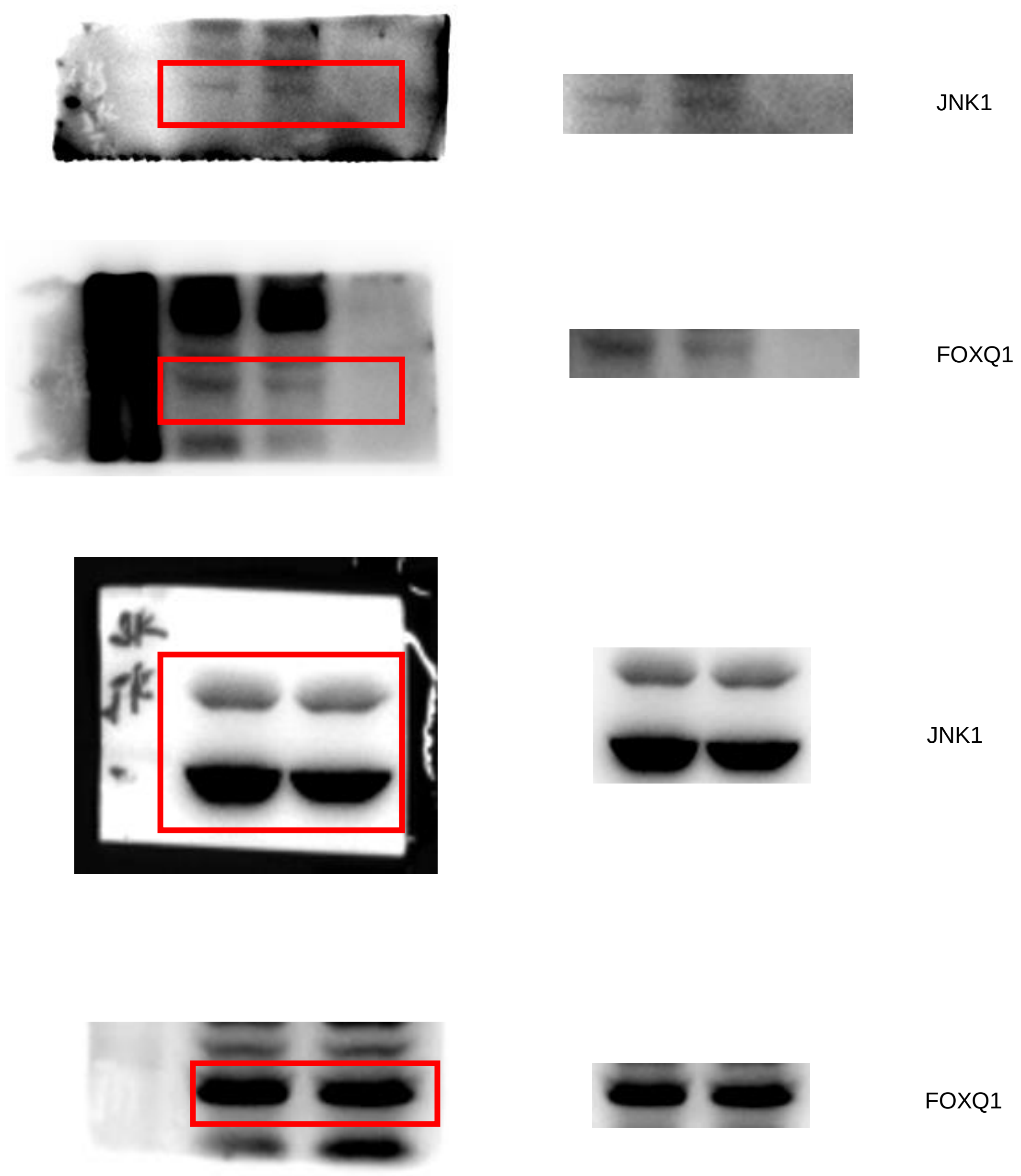

Figure 5E PLC/PRF/5

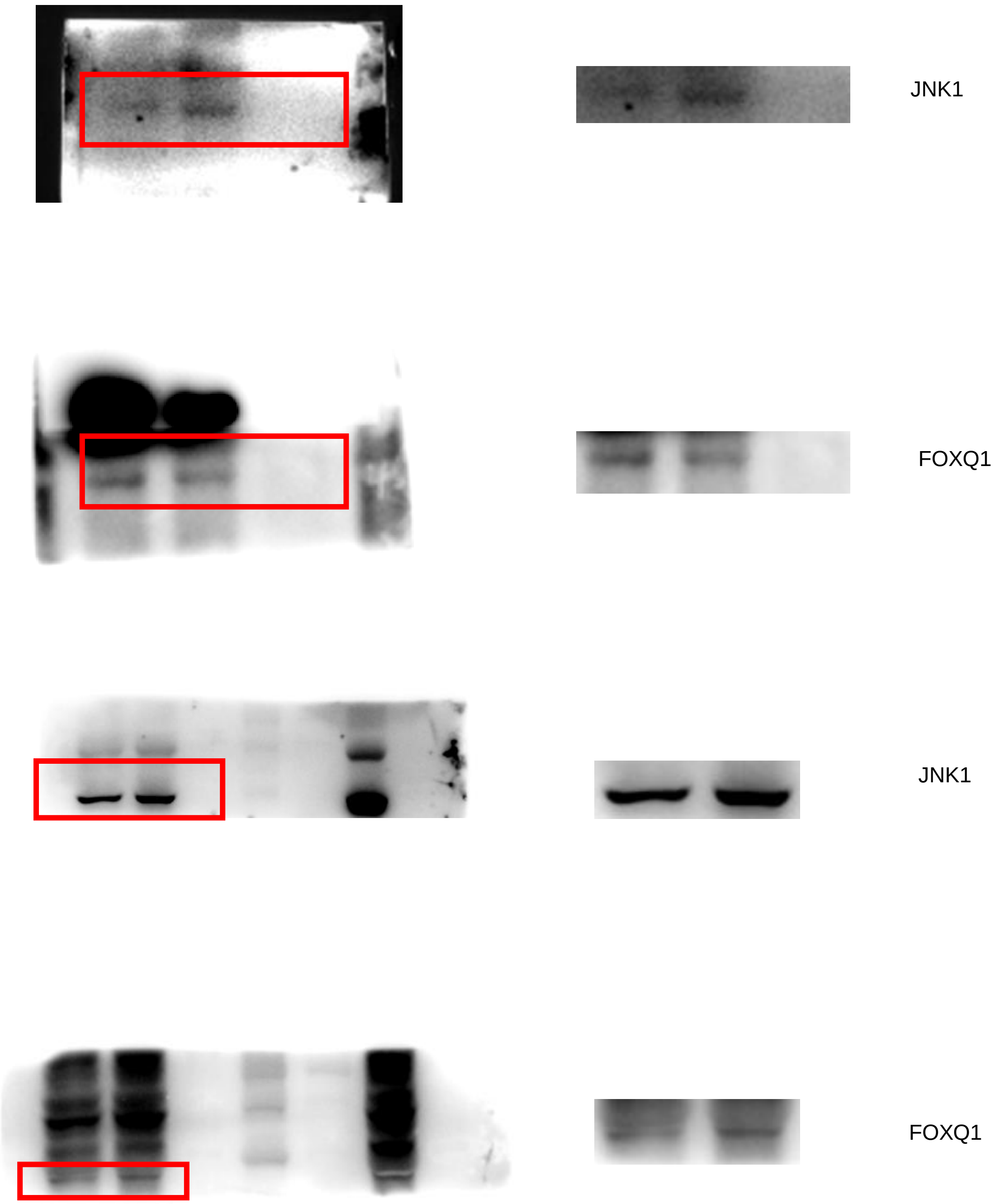

Figure 6E SK-Hep1

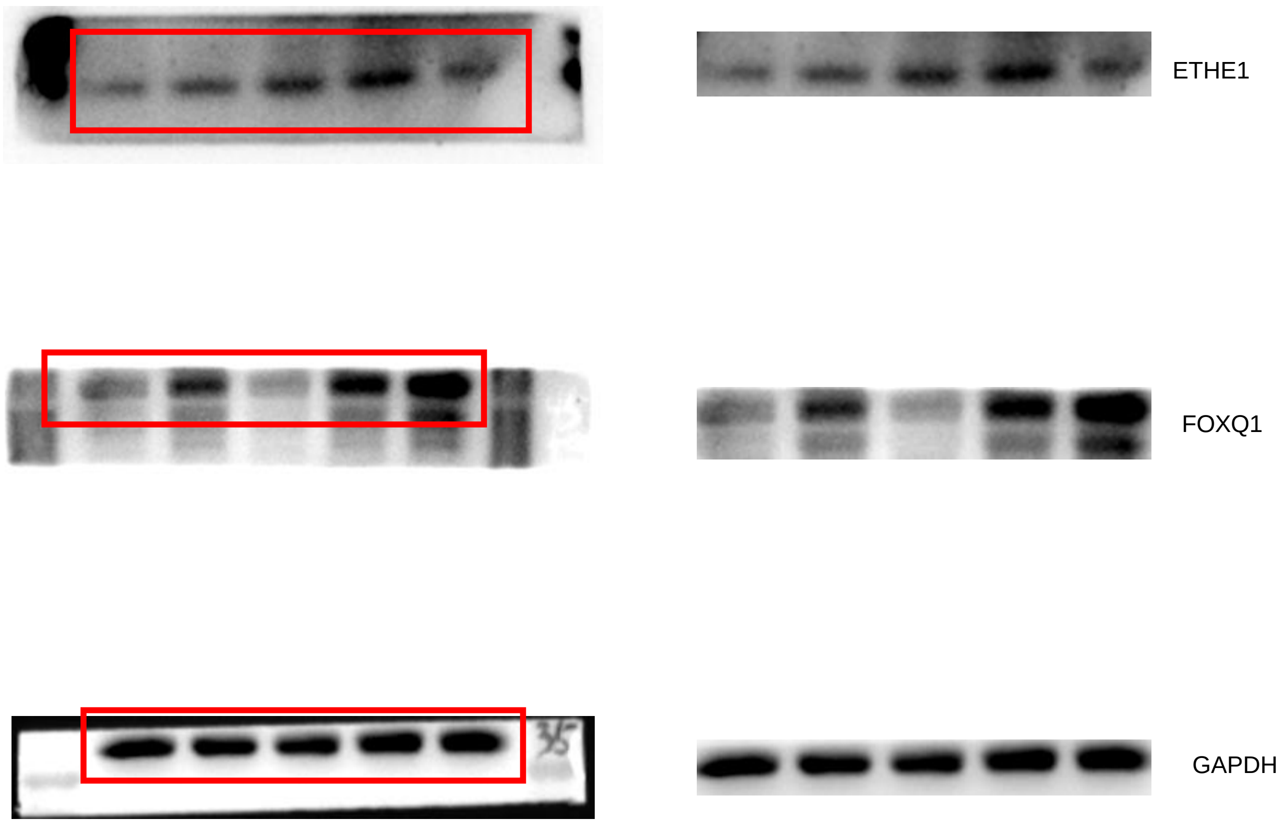

Figure 6E PLC/PRF/5

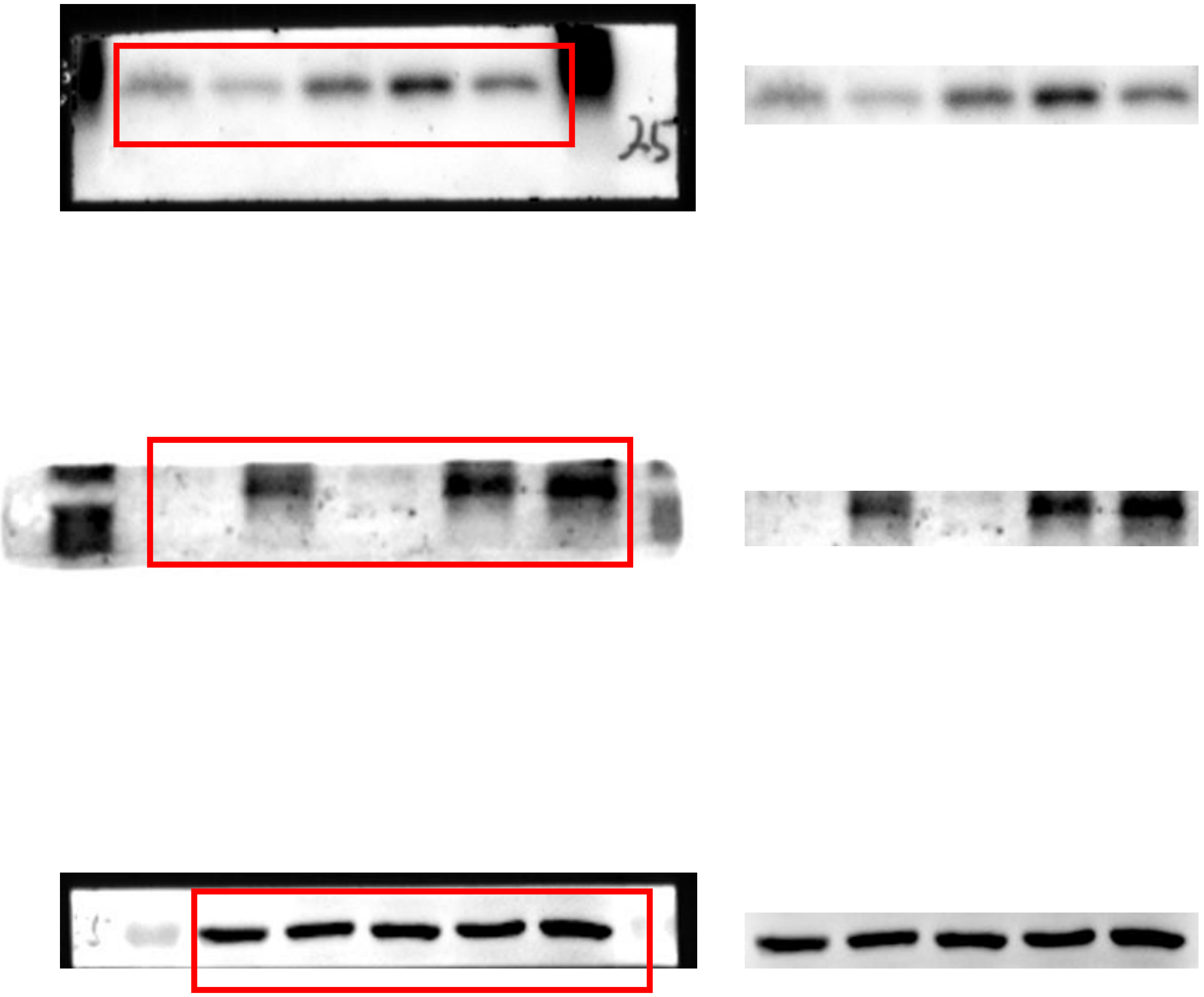

ETHE1

FOXQ1

GAPDH

Figure 7D

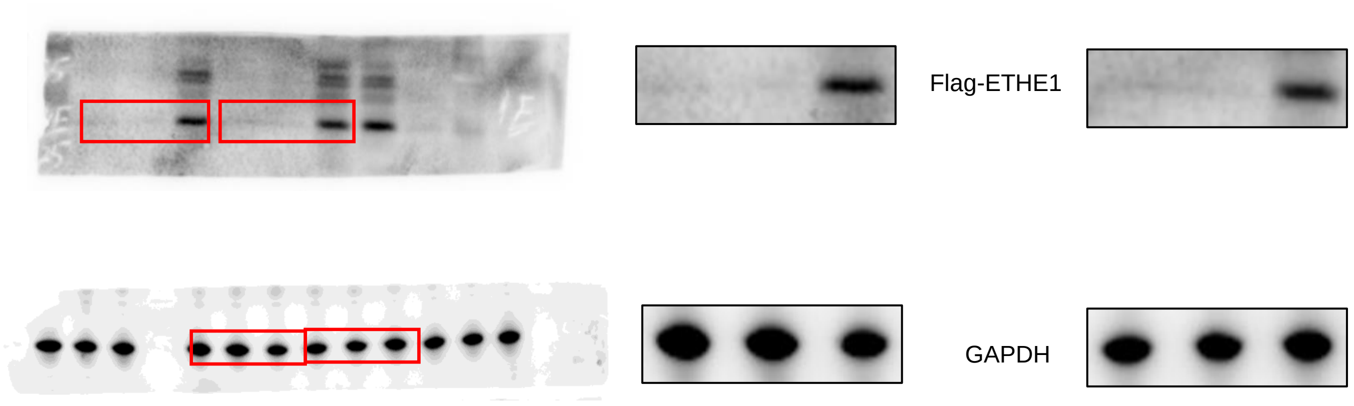

Figure 7F

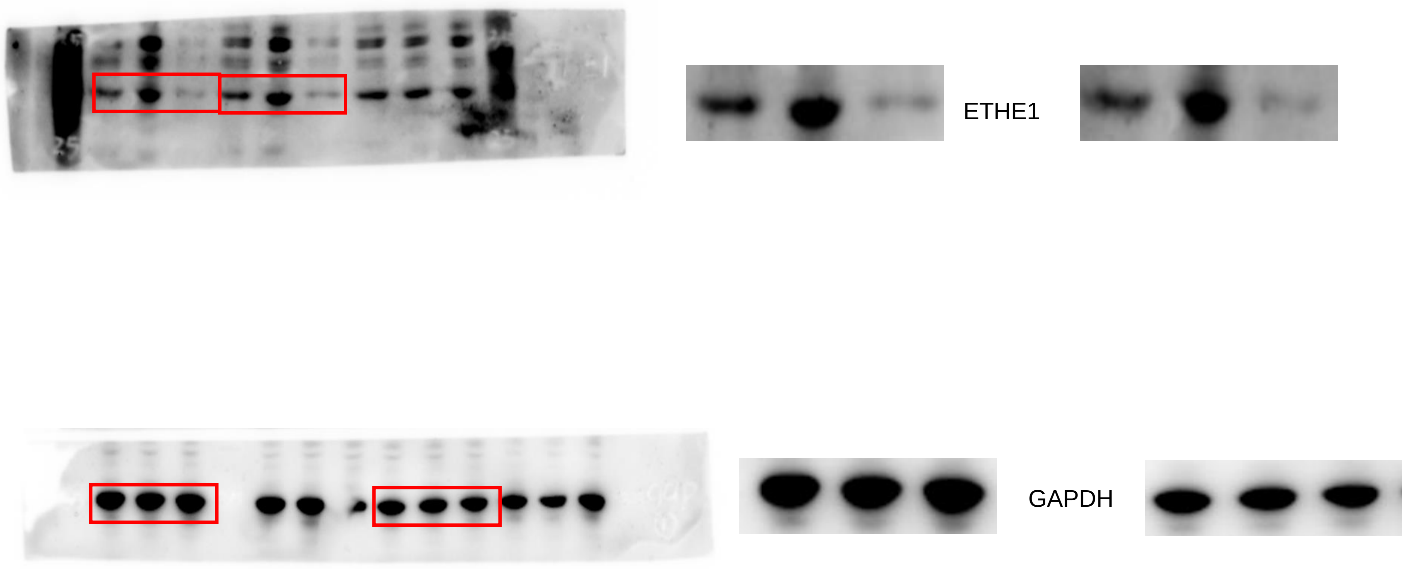

Figure S2 B

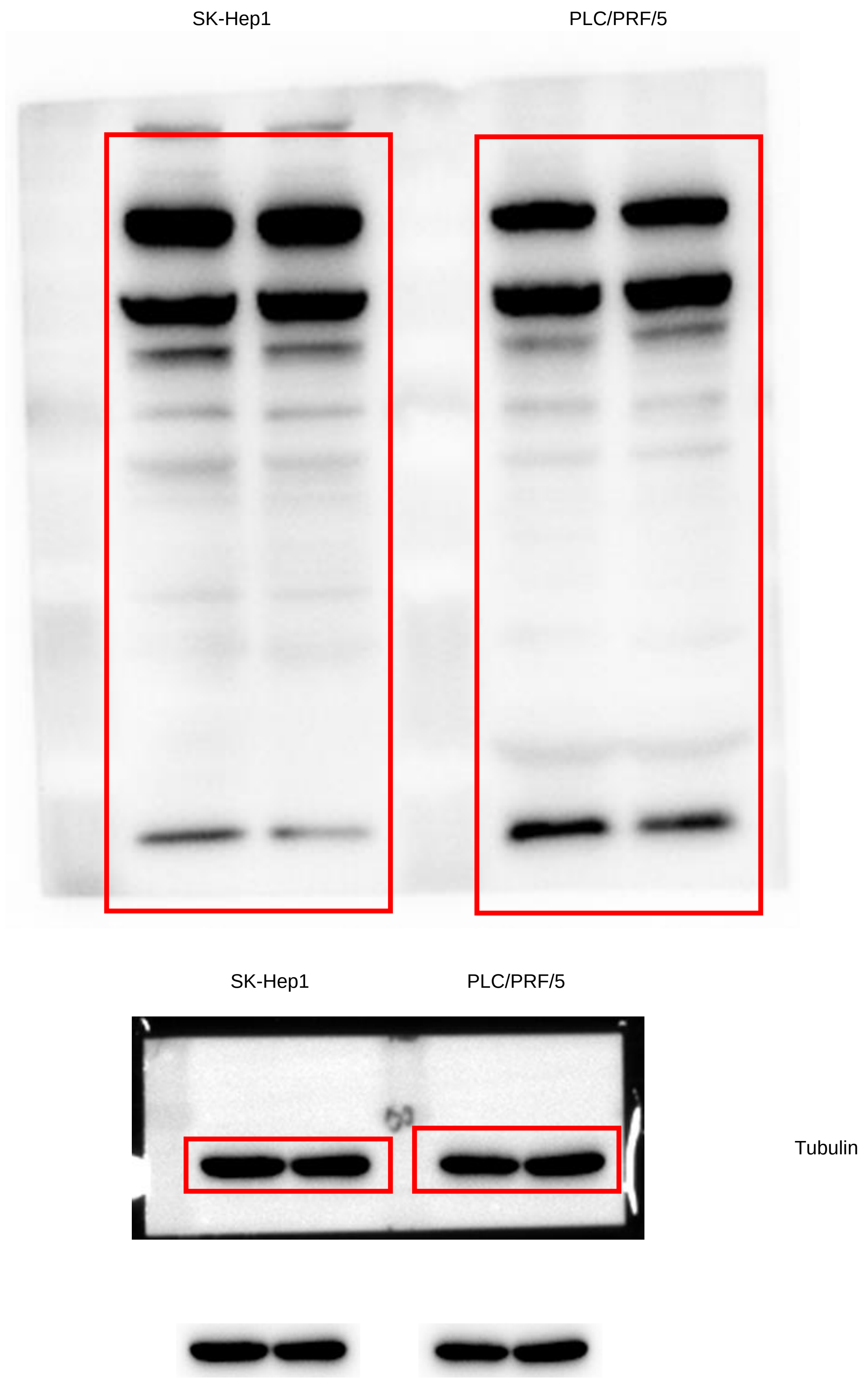

Figure S2 D

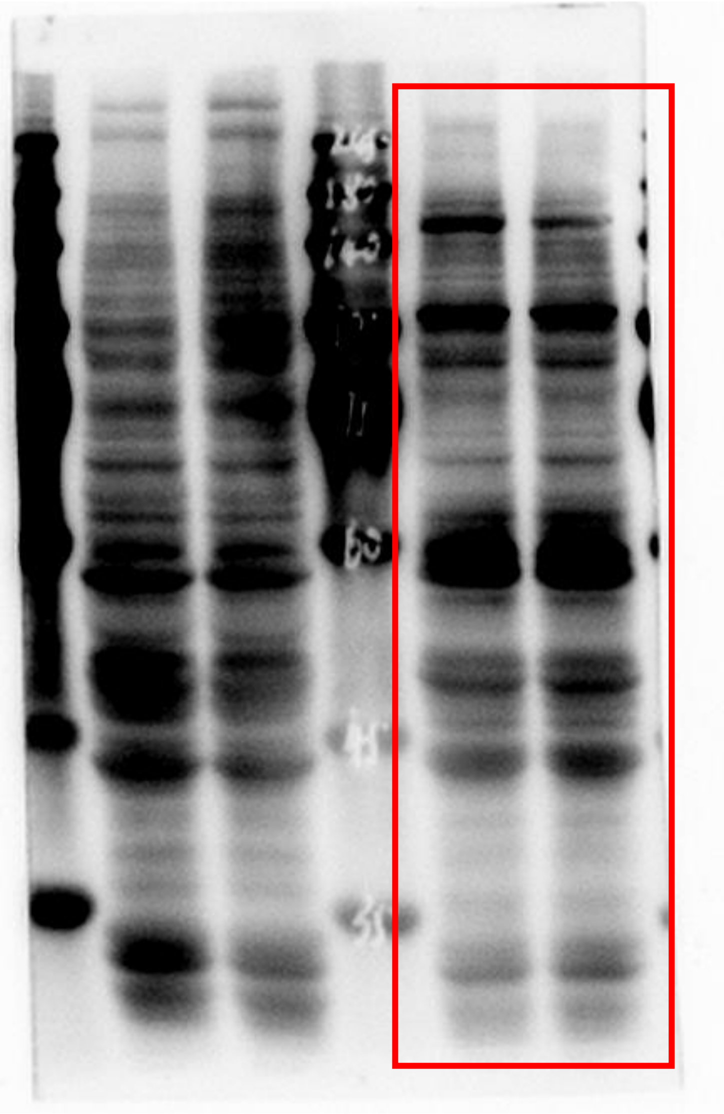

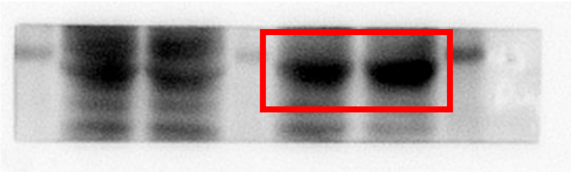

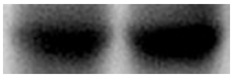

IB:Flag

Figure S2 E

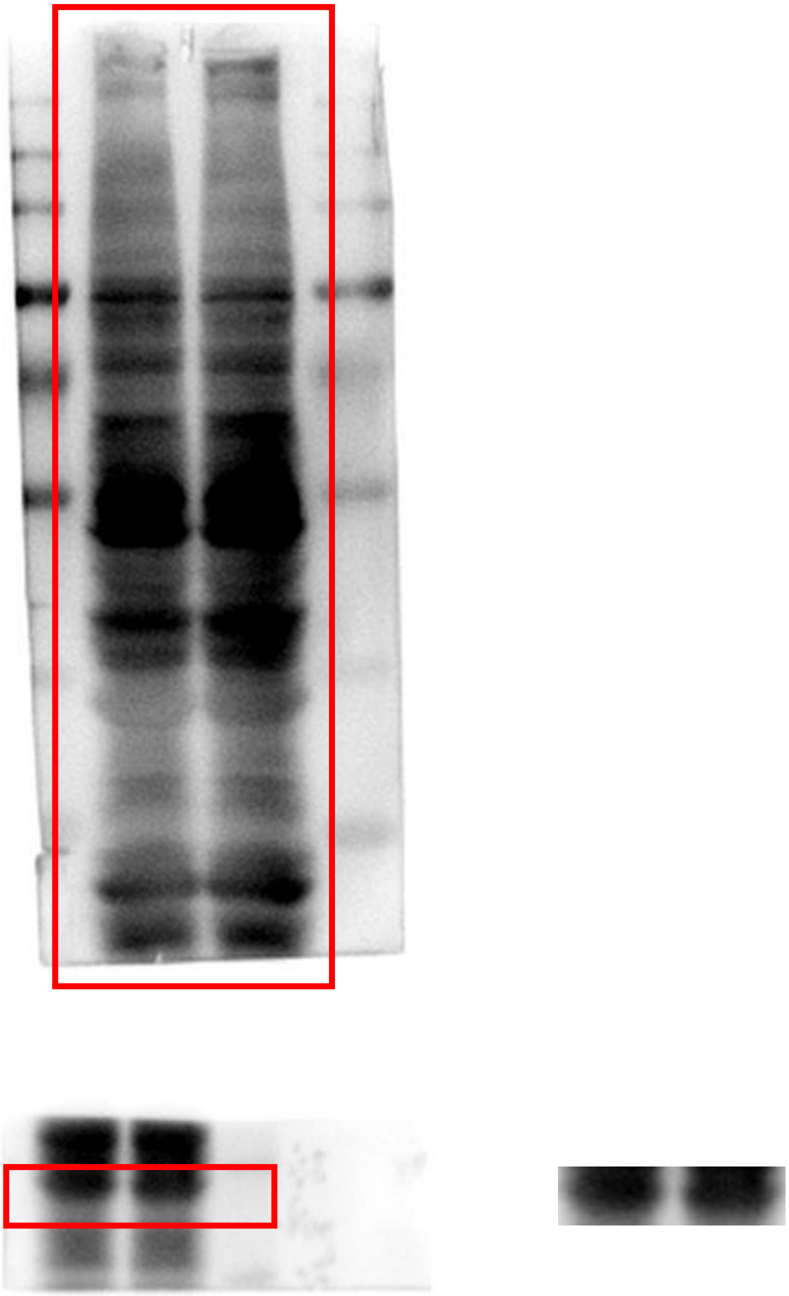

IB:Flag

Figure S3 A

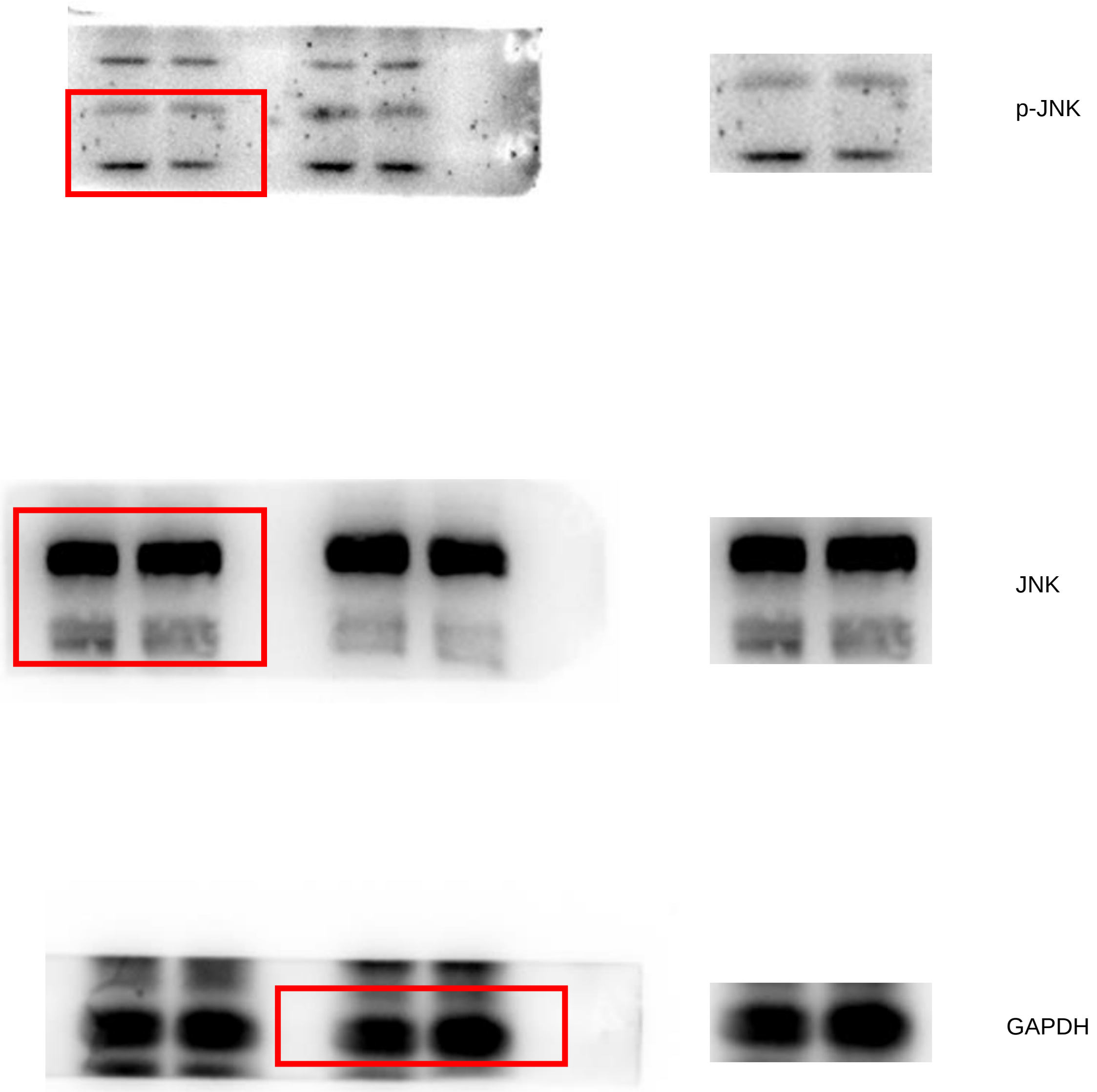

Figure S3 B

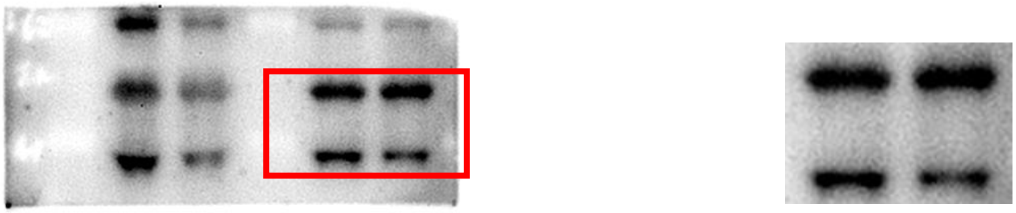

p-JNK

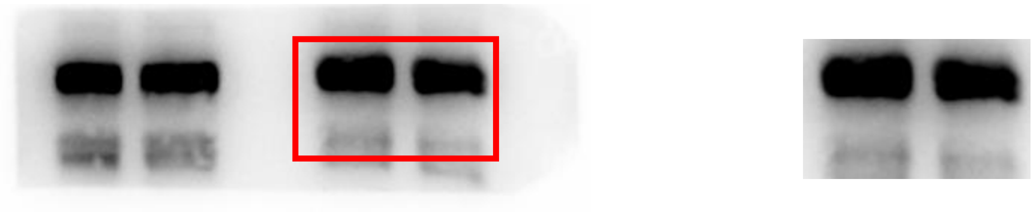

JNK

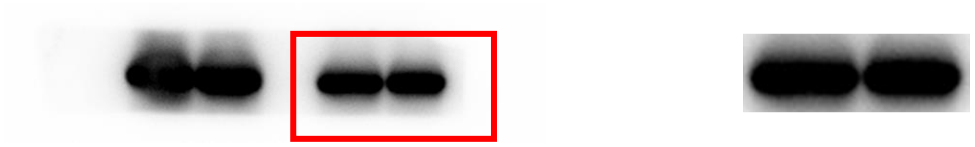

GAPDH

Figure S3 C

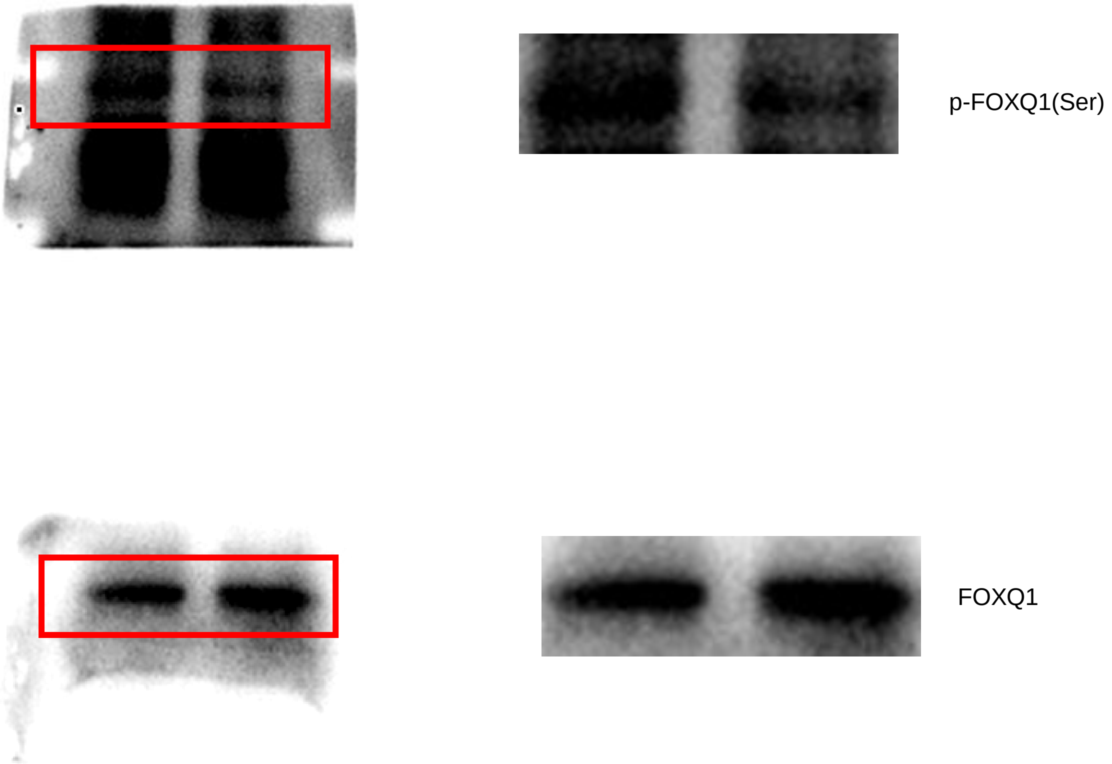

Figure S3 D

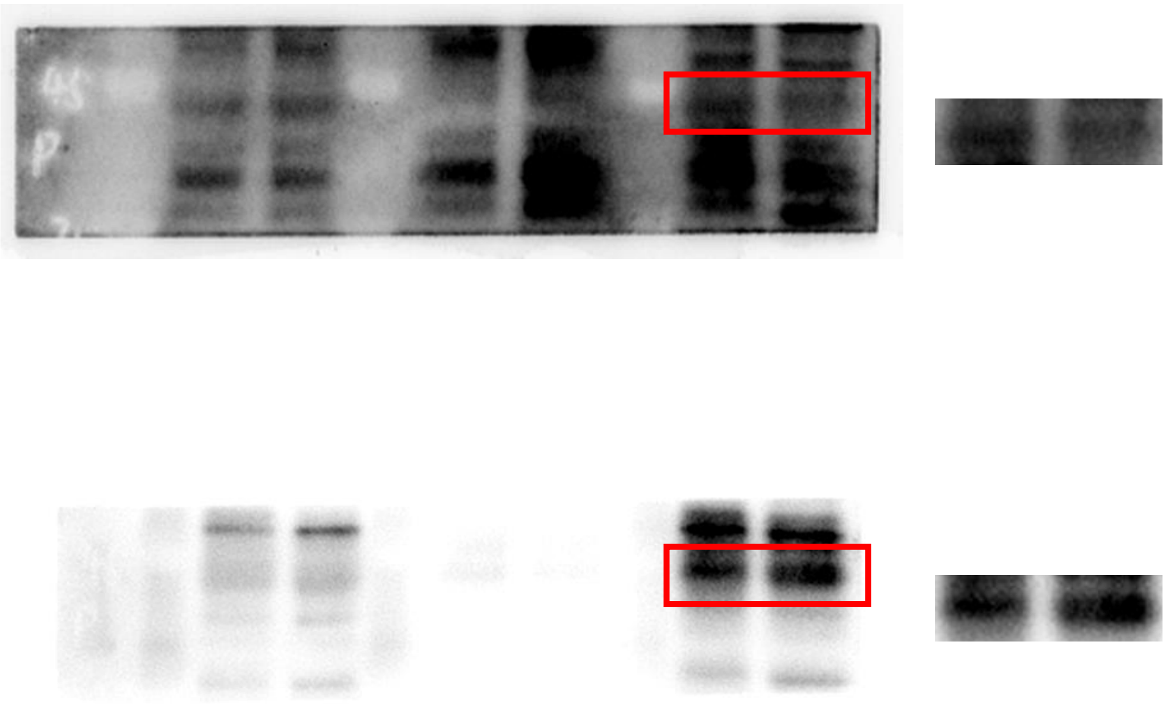

p-FOXQ1(Ser)

FOXQ1

Figure S4 A

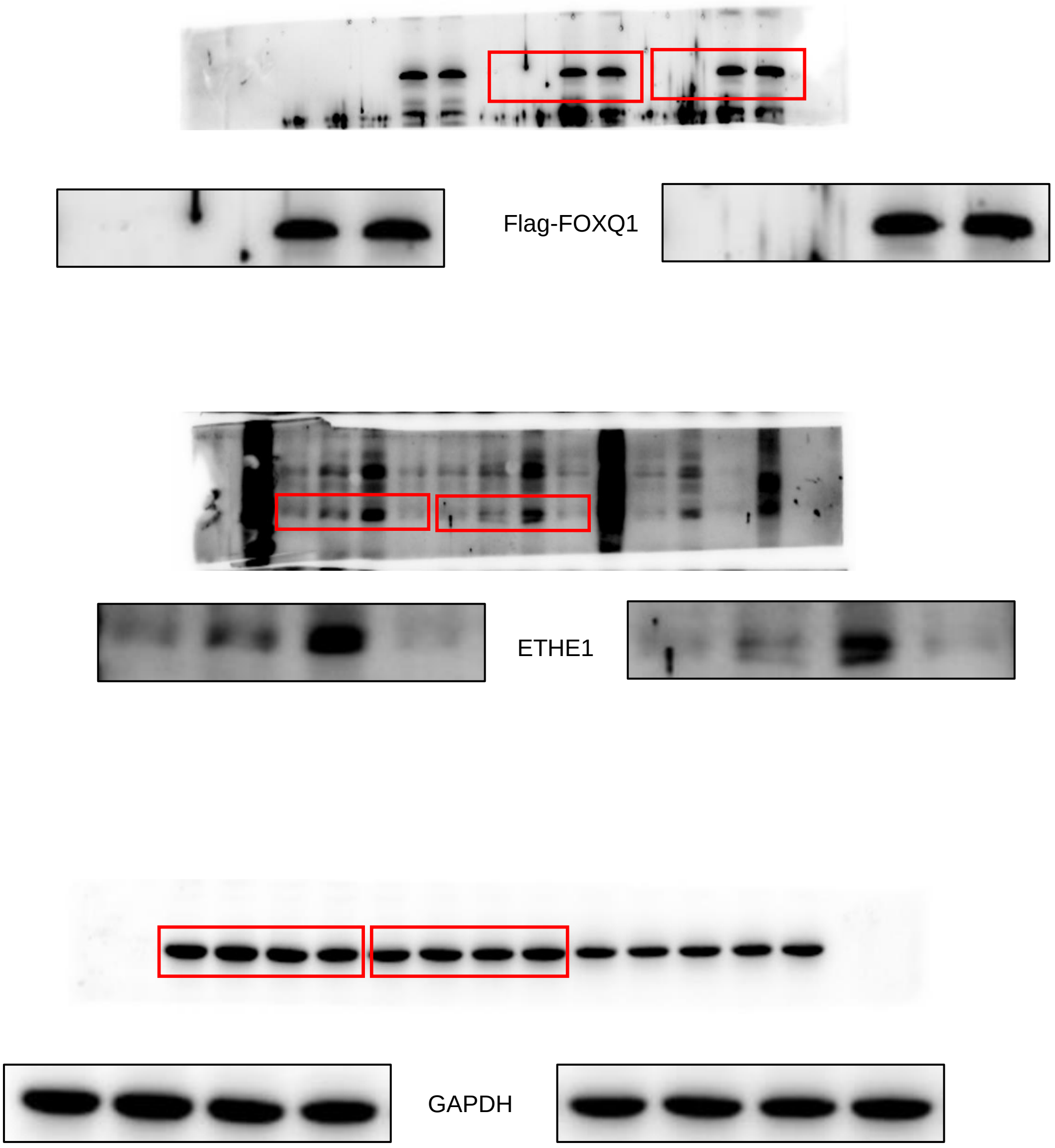

Figure S4 B

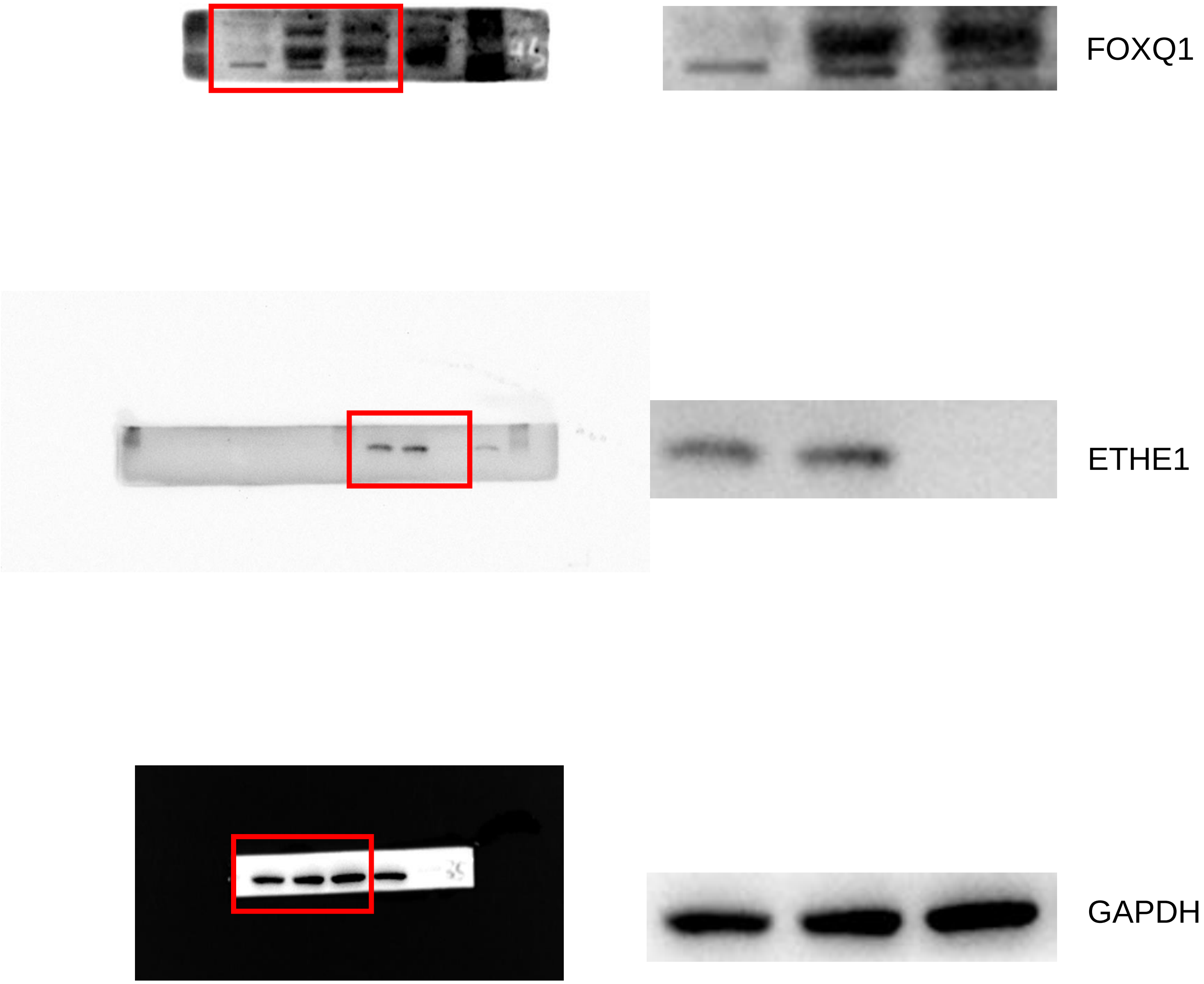

Supplement: Supplementary file 2 — Original western blots [file 41419_2024_6789_MOESM2_ESM.pdf]
